# HDAC7 promotes NSCLC proliferation and metastasis via stabilization by deubiquitinase USP10 and activation of β-catenin-FGF18 pathway

**DOI:** 10.1186/s13046-022-02266-9

**Published:** 2022-03-11

**Authors:** Kai Guo, Zhiqiang Ma, Yujiao Zhang, Lu Han, Changjian Shao, Yingtong Feng, Fei Gao, Shouyin Di, Zhipei Zhang, Jiao Zhang, Fabrizio Tabbò, Simon Ekman, Kenichi Suda, Federico Cappuzzo, Jing Han, Xiaofei Li, Xiaolong Yan

**Affiliations:** 1 Department of Thoracic Surgery, Shaanxi Provincial People’s Hospital, The Third Affiliated Hospital of Xi’an Jiaotong University, Xi’an, 710068 China; 2 Department of Thoracic Surgery, Tangdu Hospital, Air Force Medical University, 1 Xinsi Road, Xi’an , 710038 China; 3 Department of Medical Oncology, Senior Department of Oncology, The Fifth Medical Center of PLA General Hospital, 8 Dongdajie Road, Beijing, 100071 China; 4 Department of Respiratory and Critical Care Medicine, the Second Affiliated Hospital of Xi’an Jiaotong University, 157 Xiwu Road, Xi’an, 710003 China; 5 Department of Ultrasound, Xi’an Central Hospital, Xi’an Jiaotong University, 161 Xiwu Road, Xi’an, 710003 China; 6 Department of Neurosurgery, Tangdu Hospital, Air Force Medical University, 1 Xinsi Road, Xi’an, 710038 China; 7 Department of Thoracic Surgery, The Sixth Medical Center of PLA General Hospital, 6 Fucheng Road, 100048 Beijing, China; 8 Department of Oncology, University of Turin, San Luigi Hospital, Orbassano, TO Italy; 9 Thoracic Oncology Center, Department of Oncology-Pathology, Karolinska University Hospital, Karolinska Institutet, Stockholm, Sweden; 10 Division of Thoracic Surgery, Department of Surgery, Kindai University Faculty of Medicine, 377-2 Ohno-higashi, Osaka-Sayama, 589-8511 Japan; 11 Istituto Nazionale Tumori IRCCS “Regina Elena”, via Elio Chianesi 53, 00144 Roma, Italy; 12 Department of Ophthalmology, Tangdu Hospital, Air Force Medical University, 1 Xinsi Road, Xi’an, 710038 China

**Keywords:** NSCLC, HDAC7, FGF18, β-catenin, USP10

## Abstract

**Background:**

Histone deacetylases (HDACs) play crucial roles in cancers, but the role and mechanism of HDAC7 in NSCLC have not been fully understood.

**Methods:**

A total of 319 patients with non-small cell lung cancer (NSCLC) who underwent surgery were enrolled in this study. Immunohistochemistry and Kaplan–Meier survival analysis were performed to investigate the relationship between HDAC7, fibroblast growth factor 18 (FGF18) expression, and clinicopathologic characteristics. Cell functional experiments were implemented both in vivo and in vitro to investigate the effects on NSCLC cell proliferation and metastasis. Recombinant lentivirus–meditated in vivo gene overexpression or knockdown, real-time polymerase chain reaction (PCR), western blotting, and coimmunoprecipitation assays were applied to clarify the underlying molecular mechanism of HDAC7 in promoting NSCLC progression.

**Results:**

The elevated expression of HDAC7 or FGF18 was positively correlated with poor prognosis, tumor–node–metastasis (TNM) stage, and tumor differentiation of NSCLC patients. NSCLC patients with co-expressed HDAC7 and FGF18 suffered the worst prognosis. HDAC7 overexpression promoted NSCLC proliferation and metastasis by upregulating FGF18. Conversely, overexpression of FGF18 reversed the attenuated ability in tumor growth and metastasis mediated by downregulating HDAC7. In terms of mechanism, our results suggested that the interaction of HDAC7 with β-catenin caused decreased β-catenin acetylation level at Lys49 and decreased phosphorylation level at Ser45. As a consequence, the HDAC7-mediated posttranslational modification of β-catenin facilitated nuclear transfer and activated FGF18 expression via binding to TCF4. Furthermore, deubiquitinase USP10 interacted with and stabilized HDAC7. The suppression of USP10 significantly accelerated the degradation of HDAC7 and weakened NSCLC growth and migration.

**Conclusions:**

Our findings reveal that HDAC7 promotes NSCLC progression through being stabilized by USP10 and activating the β-catenin-FGF18 pathway. Targeting this novel pathway may be a promising strategy for further developments in NSCLC therapy.

**Supplementary Information:**

The online version contains supplementary material available at 10.1186/s13046-022-02266-9.

## Background

Worldwide, lung cancer remains the leading cause of cancer incidence and mortality [1]. Although great progress has been achieved in prevention, diagnosis and treatment in recent years, the long-term prognosis of non-small cell lung cancer (NSCLC) patients remains poor because of fast proliferation and frequent metastasis. Therefore, the molecular mechanisms underlying NSCLC malignant progression, which remains not fully understood, need to be further clarified to promote therapeutic strategies.

Histone deacetylases (HDACs) are a family of total eighteen proteins that are grouped into classes I–IV based on their homology to respective yeast orthologues. The HDACs family members are key regulators in transcription, metabolism, differentiation, and angiogenesis [2]. Recent studies have indicated that the aberrant expression of HDACs proteins plays vital roles in tumorigenesis and progression. Among the four classes of HDACs family, HDAC IIa subgroup contains four members: HDAC4, HDAC5, HDAC7, and HDAC9. Apart from their involvement in (auto)immune- and neurological disorders, diabetes, and muscle degenerative diseases [3], the role of these proteins in cancers attracted great attention. HDAC4 has been extensively revealed to serve an important role in tumor invasion and metastasis in a variety of tumors, such as colorectal cancer, lung cancer and multiple myeloma [4–6]. HDAC5 overexpression led to growth suppression and apoptosis by activating tumor necrosis factor death receptor pathway in osteogenic sarcoma, neuroblastoma and breast cancer [7]. HDAC9 has been reported to be up-regulated in breast cancer and retinoblastoma [8, 9]. Previously, we have suggested that HDAC9 downregulation mediated the anti‐NSCLC actions of melatonin [10]. HDAC7, however, has a crucial role in regulating cell proliferation, migration and apoptosis in pathophysiological processes [11]. Aberrant expression of HDAC7 has been observed in gastric, breast, and ovarian cancer, along with glioma and hematological malignancies [12–16], where high expression of HDAC7 has been correlated with metastasis and poor prognosis [12, 16]. HDAC7 has also shown antitumor effects in certain tumors, such as pro-B acute lymphoblastic leukemia (pro-B-ALL) and Burkitt lymphoma [17]. Although a few previous studies have indicated that HDAC7 was involved in tumorigenesis and cell growth in lung cancer [18, 19], the expression and mechanisms underlying the oncogenic function in NSCLC remains largely unknown. For potential clinical implementation and future drug development, the exact role and detailed mechanism of action of HDAC7 in NSCLC need to be further investigated.

In the present study, the role of HDAC7 in NSCLC proliferation and metastasis was investigated. We demonstrated that HDAC7 acts as an oncogenic protein through regulation of the FGF18 expression. We then found that HDAC7 activated FGF18 expression by deacetylating β-catenin at Lys49 and facilitating β-catenin nuclear redistribution. Moreover, HDAC7 could be deubiquitinated and stabilized by USP10. These results may provide the theoretical and experimental basis for further development of NSCLC therapy.

## Materials and methods

### Patients and materials

In this retrospective study were included 319 NSCLC patients who had not previously received radiotherapy, chemotherapy, or targeted therapy and who had been diagnosed and treated surgically at the Department of Thoracic Surgery of Tangdu Hospital of Air Force Medical University (Xi’an, China) between May 2009 and January 2014.

The study was approved by the ethics committee of the hospital (ethical approval number 202003–019). Written informed consent was obtained from all patients before any study-related procedures began. The complete follow-up was updated until death or January 2019, whichever came first. Furthermore, 12 pairs of frozen NSCLC tissues and corresponding tumor-adjacent normal tissues were randomly selected for western blot analysis.

### Tissue samples of NSCLC patients, tissue microarray, and immunohistochemistry

The 319 pairs of formalin-fixed NSCLC tissues and corresponding tumor-adjacent normal tissues were placed into a paraffin-embedded tissue microarray (TMA). Immunohistochemistry **(**IHC) staining was carried out on TMA sections according to standard practice, using the primary antibodies of anti-HDAC7 (1:100, #33418, Cell Signaling Technology (CST)) and anti-FGF18 (1:200, 11495–1-AP, Proteintech).

The scoring of the IHC intensity included negative (score 0), weak (score 1), moderate (score 2), or strong staining (score 3). The proportion of positive stains was scored as 0 (< 5%), 1 (6%–25%), 2 (26%–50%), 3 (51%–75%), or 4 (> 75%). These 2 scores were multiplied to produce the total score. The IHC staining was read by 2 expert pathologists. Any disagreement between them was resolved by discussion with a third pathologist. All pathologists were blinded with no information of the clinical data until statistical analysis. IHC staining results with low or high levels of HDAC7 or FGF18 expression were stratified by their respective average score.

### Cell culture and lentivirus infection

Human NSCLC cell lines (H1299, A549, Calu-1, SK-LU-1) and HEK-293T were obtained from the American Type Culture Collection (ATCC)**,** and cultured in Dulbecco’s Modified Eagle Medium (DMEM; Gibco, supplemented with 10% fetal bovine serum (FBS, Biological Industries), 1% (v/v) penicillin–streptomycin solution (Hyclone). The HDAC7, FGF18, USP10 and respective control lentiviruses were all obtained from Genechem Corporation (Shanghai, China). H1299, A549, Calu-1, and SK-LU-1 cells were performed lentiviral infection according to the protocol of the Genechem Recombinant Lentivirus Operation Manual and further selected for stable cell lines.

### Analysis of cell viability

Cell viability was assessed using Cell Counting Kit-8 (CCK-8, 7Sea) following the manufacturer’s instructions. Optical density (OD) values were obtained at 450 nm using a microplate reader (SpectraMax M5, Molecular Device).

### 5-Ethynyl-2′-deoxyuridine incorporation assay

Cell proliferation was assessed by 5-ethynyl-2′-deoxyuridine **(**EdU) incorporation assay. BeyoClick EdU Cell proliferation Kit with Alexa Fluor 594 (Beyotime) was used according to manufacturer’s instructions. For confocal microscopy, cells were imaged on the Olympus FV1000 confocal microscope (Olympus). EdU-positive cells were manually counted and expressed as the percentage of cells calculated from nuclear labeling with Hoechst 33342.

### Colony formation assay

For clonogenic assays, 500 cells were seeded and cultured in the 6-well plates for 10–14 days. Then, colonies were fixed with formalin and stained with 0.1% crystal violet (Solarbio). After the plates were photographed, colony numbers were counted using the ImageJ software (National Institutes of Health).

### Scratch wound assay

Scratch wound assays were performed in 6-well plates. Once cells were 100% confluent, cells were washed once with phosphate-buffered saline (PBS) and cultured in serum-free media (SFM) for 24 h. A wound was created by scraping the cell monolayer with a p200 pipet tip and then media was immediately replaced with SFM. The digital images were taken immediately after scratching and 24 h post scratch with an inverted microscope. The areas of scratch without cells immediately after scratching and the remaining areas without cells at the end of the assay were calculated using ImageJ software.

### Transwell cell migration assay

The abilities of cell migration were quantified using transwell assays. Cell transwell chambers were obtained from Corning (Costar 3422). Cells were seeded on the upper chamber and filled with 300 μL DMEM medium without FBS, and the lower chamber was filled with 1000 μL DMEM containing 10% FBS. After 24 h, the migrated cells were fixed by formalin, stained with 0.1% crystal violet (Solarbio), photographed, and counted.

### Immunofluorescence

Cultured cells were washed once with PBS and fixed with 4% paraformaldehyde for 20 min. Fixed cells were washed 3 times with PBS for 5 min each time. Cells were then permeabilized with Triton X-100 (9002–93–1, Sigma-Aldrich) in PBS at 4 °C for 10 min and blocked with 3% bovine serum albumin (BSA) for 30 min at room temperature. Primary antibodies were diluted and incubated with the cells at 4 °C overnight. Cells were washed 3 times with PBS and incubated with fluorescent secondary antibodies at room temperature for 1 h. 4′,6-diamidino-2-phenylindole (DAPI) was added for 5 min to visualize the nuclei. The fluorescence was observed and photographed under an Olympus FV1000 confocal microscope.

### Cytoplasmic and nuclear protein extraction

The nuclear-cytosolic protein isolation kit (P0027, Beyotime) was obtained for extracting cytosolic and nuclear fractions. Cultured cells were washed twice and collected with 1 mL of PBS. The cell suspension was centrifuged in 4 °C at 90 g for 3 min. After the supernatant fraction was discarded, the sediment was resuspended in reagent A with fresh phenylmethylsulfonyl fluoride (PMSF). Samples were vortexed vigorously for 5 s and placed on ice for 15 min before reagent B was added. The samples were then centrifuged at 14,000 g for 5 min at 4 °C. The supernatant fraction containing the cytosolic proteins was carefully collected and used immediately or stored at − 80 °C. The sediment was resuspended in nuclear protein extraction reagent with PMSF and vortexed vigorously for 30 s and then placed on ice for 2 min. The last 2 steps were repeated for 30 min. The samples were centrifuged at 14,000 g for 10 min at 4 °C. The supernatant fractions containing the nuclear proteins were used immediately or stored at − 80 °C. The concentrations of the extracted proteins were measured using a BCA Protein Assay Kit (23227, Thermo Fisher Scientific).

### Western blotting

Western blotting was performed according to the procedures described previously [20], loading 25 μg of total protein lysate per lane. Primary antibodies were diluted at 1:1000 and secondary antibodies were diluted at 1:5000. Antibodies against the following proteins were used for the study: HDAC7 (#33418), USP10 (#8501), β-catenin (#8480), acetyl-β-catenin (Lys49; #9030), phospho-β-catenin (Thr41/Ser45; #9565), phospho-β-catenin (Ser552; #5651), phospho-β-catenin (Ser675; #4176), Cyclin E1 (#4129), E-cadherin (#14472), Slug (#9585), β-actin (#3700), Lamin B1 (#13435), and GAPDH (#5174) (CST). HDAC7(26207–1-AP), CDK1(19532–1-AP), CDK2 (10122–1-AP), CDK4 (11026–1-AP), CDK6 (14052–1-AP), Cyclin A2 (66391–1-Ig), Cyclin B1 (55004–1-AP), Cyclin D1 (60186–1-Ig), Cyclin D3 (26755–1-AP), FGF18 (60341–1-Ig), FGFR3 (66954–1-Ig), Snail (13099–1-AP), vimentin (10366–1-AP), ZEB1 (21544–1-AP), Flag (66008–3-Ig), and ubiquitin (10201–2-AP) were also used (Proteintech Group).

### Immunoprecipitation assays

Cultured cells were washed 2 times in PBS and lysed with EBC buffer (50 mM Tris–HCl, pH 7.5, 120 mM NaCl, 0.5% NP-40, and protease inhibitor cocktail). The whole cell lysates were collected using centrifugation at 13,000 g for 10 min at 4℃. The protein concentrations were measured using a BCA Protein Assay Kit (Thermo Fisher Scientific).

For the Flag tag immunoprecipitation assay, 1000 μg whole cell lysates were incubated with 6 μL anti-Flag M2 Affinity Gel (A2220, Sigma Aldrich) at 4 °C for 4 h on a rotating incubator. Then, the beads were washed 4 times with NETN buffer (20 mmol/L Tris, pH 8.0, 100 mmol/L NaCl, 1 mmol/L EDTA, and 0.5% NP-40). After being diluted with SDS loading buffer, the beads were boiled for subsequent western blotting. For endogenous immunoprecipitation assay, precleared lysates were incubated with 8 μL indicated immunoprecipitation antibody overnight at 4 °C, followed by the addition of 40 μL of prewashed PuroProteome Protein G Magnetic Beads (LSKMAGG02, Millipore) for 2 h at 4 °C. Beads were washed 4 times with NETN buffer, mixed with SDS loading buffer, and boiled. The supernatant fractions were then subjected to western blotting according to standard protocols. Normal mouse immunoglobin G (IgG) or rabbit IgG (Millipore) were used as the negative control.

### RNA sequencing and pathway enrichment analysis

Total RNA was extracted from control and HDAC7 overexpression groups of SK-LU-1 cells using TRIzol reagent (Invitrogen). Each group was prepared with 3 parallel replicates. Further RNA sequencing (RNA-seq) detection and analysis were conducted using BGISEQ-500 platform (BGI Corporation). The pathway analysis for differentially expressed genes (DEGs) was performed based on the Gene Ontology (GO) database. The Dr. Tom online platform of BGI was used for data analysis (http://report.bgi.com). The raw RNA-seq data has been uploaded on to the NCBI database (accession number: PRJNA786842).

### Real-time quantitative polymerase chain reaction

Total RNA was harvested from cells with TRIzol reagent (Invitrogen). Complementary DNA was generated using a Prime Script RT Master Mix (TaKaRa). Quantitative real-time polymerase chain reaction (qRT-PCR) was performed with SYBR Premix Ex Taq II (TaKaRa) to detect targeted messenger RNA (mRNA) levels, and data were analyzed with the MxPro software (Stratagene). GAPDH expression was used as an internal control. The primers were obtained from Genechem Corporation. The primer sequence was as follows:

- HDAC7-F: GAAAGAACAGTCCATCCCAACA
- HDAC7-R: GCTTATAGCGCAGCTTCAGG
- c-Myc-F: GGCTCCTGGCAAAAGGTCA
- c-Myc-R: CTGCGTAGTTGTGCTGATGT
- MMP7-F: GAGTGAGCTACAGTGGGAACA
- MMP7-R: CTATGACGCGGGAGTTTAACAT
- GAPDH-F: TGACTTCAACAGCGACACCCA
- GAPDH-R: CACCCTGTTGCTGTAGCCAAA

### TOP/FOP flash activity assay

SK-LU-1 and A549 Cells were seeded into 6-well plates for further experiments. TCF-responsive promoter reporter (TOP-flash) or nonresponsive control reporter (FOP-flash) β-catenin firefly luciferase reporter gene constructs, and a pRL-SV40 Renilla luciferase construct were transfected with Lipofectamine 2000 (Invitrogen; Thermo Fisher Scientific, Inc). The pRL-SV40 Renilla luciferase construct was used as the internal control. Later, Dual-Luciferase Reporter Assay System (Promega Corporation) were used to detect the luciferase activity of TOP and FOP. Relative ratio of TOP and FOP luciferase activity was represented as the mean ± standard error of mean after normalizing to the control.

### Enzyme-linked immunosorbent assay

The enzyme-linked immunosorbent assays (ELISA) were performed using the ELISA Kit for Human FGF18 (Cloud-clone Corp, Sec907Hu, Wuhan, China). Briefly, NSCLC cells overexpressing or silencing HDAC7 or the control were cultured for 48 h. Cell-free supernatant were harvested and FGF18 concentrations were quantified with the ELISA Kits respectively according to the manufacturer’s instructions.

### Flow cytometry

A549 cells synchronized with serum starvation for 48 h and then released back into the cell cycle were collected at the indicated time point. Cells were washed three times with PBS, centrifuged (1200 g) for 10 min, and resuspended in 75% ethanol at 4 °C overnight. Then cells were washed again with PBS, resuspended and stained with propidium iodide (PI) and RNaseA (C1052, Beyotime, China) according to the manufacturer’s instructions. The cell cycle was then analyzed by flow cytometry on a FACSCalibur flow cytometer (BD Biosciences, USA).

### Animal experiments

All animal experiments were approved by the Animal Care Committee of Air Force Medical University. Male athymic nude mice (6–8 weeks, 18–20 g) were obtained from the Laboratory Animal Center of the university. Mice were kept under a 12-h light–dark cycle. Temperatures of 65–75 °F (18–23 °C) with 40–60% humidity were also used as housing conditions for the mice.

### In vivo tumor xenograft assay and lung metastasis assay

Nude mice were randomly divided into groups (5 mice per group) and received respective treatments. Briefly, for in vivo xenograft assay, different groups of cells (5 × 10^6^ in 200 µL PBS) were separately and subcutaneously inoculated into the nude mice. The body weight of each mice was measured every 3 days for 21 days. Tumors were then excised from the sacrificed mice for additional analysis.

For tail vein metastasis assay, 5 × 10^6^ cells resuspended in 100 µL of PBS were injected in the lateral tail vein of nude mice. After 4 weeks, an IVIS 100 Imaging System (Xenogen) was used for live animal imaging and acquiring photographic images of in vivo metastases of each nude mice. The mice were then sacrificed. Their lungs were dissected and prepared for standard histological examination. The resulting grayscale photographic and pseudo-colored luminescent images were automatically superimposed using IVIS Living Image software (Xenogen).

### Statistical analysis

All experiments included were repeated at least 3 times. The quantitative data were compared between groups using the *t*-test. Categorical data were analyzed using the χ2-test or Fisher’s exact test. The overall survival rates were determined using the Kaplan–Meier method and log-rank test. Univariate or multivariate survival analysis was carried out using the Cox proportional hazards model. A value of *P* < 0.05 was considered to be significant. SPSS 24.0 software (IBM Corp) was used to analyze the data.

## Results

### HDAC7 is upregulated in NSCLC, and high HDAC7 expression predicts poor survival of NSCLC patients

**Fig. 1:**
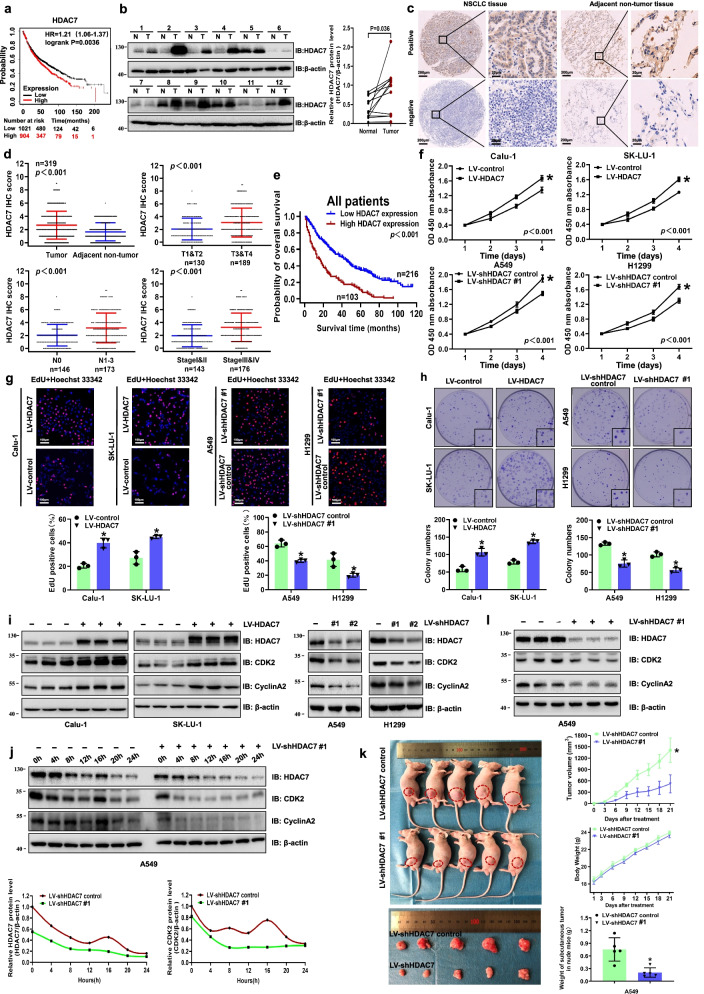
High HDAC7 expression was correlated with poor NSCLC prognosis, and HDAC7 overexpression promoted NSCLC proliferation. **a** Kaplan–Meier survival analysis of HDAC7 expression from the Kaplan–Meier plotter database. **b** Western blots of HDAC7 expression in tumor (T) and paired adjacent nontumor (N) tissues from 12 NSCLC patients. **c** Representative HDAC7 IHC images of NSCLC and adjacent non-tumor tissues. Scale bar, 200 μm and 20 μm (inset), respectively. IHC analysis (**d**) and Kaplan–Meier survival analysis (**e**) of HDAC7 expression in 319 NSCLC patients. **f** The growth curve of indicated NSCLC cells. **g** Representative images and analysis of EdU incorporation assay. EdU-positive cells (red) and Hoechst 33342–stained cells (blue). Scale bar, 100 μm (inset). **h** Representative images and analysis of colony formation assay. **i** Representative western blotting of HDAC7, CDK2 and Cyclin A2 in indicated NSCLC cells. **j** Representative western blotting of whole cell lysates (WCL) at indicated times. A549 cells were synchronized in G0/G1 by serum starvation followed by serum re-addition. **k** Gross photograph of subcutaneous xenograft tumors, tumor growth curve, changes of nude mice body weight, and subcutaneous tumor weight in each group. **l** Representative western blotting of HDAC7, CDK2, and Cyclin A2 in subcutaneous tumor tissues. All the data are expressed a mean ± SD. *P < 0.05. LV, lentivirus

In order to investigate HDAC7 expression in NSCLC, we firstly analyzed 1925 NSCLC cases in the Kaplan–Meier plotter database (http://KMplot.com). Using the Kaplan–Meier method, we found that high expression of HDAC7 was significantly correlated with poor prognosis of NSCLC patients (Fig. 1a). We then measured HDAC7 protein expression in 12 paired NSCLC and adjacent normal tissues by western blotting. We found that out of the 12 patients, 8 (66.7%) showed higher HDAC7 levels in tumors than matched normal tissues while 4 (33.3%) did not. Further statistical analysis indicated that HDAC7 expression in tumors (T) was significantly higher than that in normal tissues (N) (*P* = 0.036, Fig. 1b).

To validate these findings, we analyzed the protein expression and clinical significance of HDAC7 by IHC staining in a TMA containing 319 paired tumor–normal tissue samples (Fig. 1c); 32% (103/319) NSCLC sections were categorized as high HDAC7 expression while 17% (54/319) corresponding adjacent normal tissue sections were categorized as high HDAC7 expression. The HDAC7 levels were significantly upregulated in NSCLC tissues compared to those in adjacent normal tissues (Fig. 1d). Furthermore, the NSCLC patients with deep tumor invasion and/or larger tumors (T3/T4), high American Joint Committee on Cancer (AJCC) 8th stage (stage III–IV) and lymph node metastasis (N1–N3) had higher HDAC7 expression (Fig. 1d). The upregulated HDAC7 expression was positively correlated to larger tumor size, T stage, lymphatic invasion, distant metastasis, tumor–node–metastasis (TNM) stages, and tumor differentiation (Table 1).

**Table 1:** Correlation of HDAC7 and FGF18 expression with clinicopathological characteristics of NSCLC patients

Clinicopathological Variables	n	Tumor HDAC7 expression		*P* value	Tumor FGF18 expression		*P* value
		**Low**	**High**		**Low**	**High**	
**Age**							
≥ 60	174	**119**	55	0.776	104	70	0.586
< 60	145	**97**	48		91	54	
**Sex**							
Female	61	**41**	20	0.926	37	24	0.933
Male	258	**175**	83		158	100	
**Smoking history**							
Never	103	**68**	35	0.655	61	42	0.630
ever	216	**148**	68		134	82	
**Maximal tumor size**							
< 5 cm	118	**89**	29	0.024	82	36	0.019
≥ 5 cm	201	**127**	74		113	88	
**T stage**							
T1-T2	130	**103**	27	< 0.001	94	36	0.001
T3-T4	189	**113**	76		101	88	
**Lymphatic invasion**							
N0	146	**110**	36	0.007	103	43	0.002
N1-N3	173	**106**	67		92	81	
**Distant metastasis**							
No	309	**214**	95	0.001	192	117	0.040
Yes	10	**2**	8		3	7	
**TNM stages**							
I	47	**44**	3	< 0.001	35	12	< 0.001
II	96	**71**	25		76	20	
III	166	**99**	67		81	85	
IV	10	**2**	8		3	7	
**Tumor differentiation**							
High and moderate	219	**161**	58	0.001	145	74	0.006
poor	100	**55**	45		50	50	

Kaplan–Meier analysis showed that NSCLC patients with high HDAC7 protein expression were correlated with poor overall survival (Fig. 1e), whereas patients with low HDAC7 expression had a longer median survival time (Table 2). Cox regression analyses showed that HDAC7 was an independent prognostic factor for NSCLC patients (Table 3).

**Table 2:** Univariate analysis of the correlation between clinicopathological variables and survival of patients with NSCLC

Clinicopathological variables	Median survival time (month)	Univariate analysis		
		**HR**	**95% CI**	*P* ** value**
**Age**		1.101	0.861–1.408	0.442
≥ 60	24.00 ± 2.931			
< 60	34.00 ± 4.604			
**Sex**		1.126	0.822–1.542	0.459
Female	34.00 ± 7.809			
Male	27.00 ± 3.153			
**Smoking history**		0.964	0.742–1.252	0.783
Never	30.00 ± 4.150			
ever	27.00 ± 3.833			
**Maximal tumor size**		1.583	1.221–2.052	0.001
< 5 cm	42.00 ± 5.431			
≥ 5 cm	19.00 ± 2.255			
**T stage**		1.986	1.534–2.570	< 0.001
T1-T2	49.00 ± 5.067			
T3-T4	18.00 ± 1.874			
**Lymphatic invasion**		1.662	1.293–2.135	< 0.001
N0	42.00 ± 6.041			
N1-N3	23.00 ± 2.505			
**Distant metastasis**		2.193	1.162–4.137	0.015
No	28.00 ± 3.102			
Yes	11.00 ± 5.534			
**TNM stages**		2.897	2.234–3.756	< 0.001
I-II	54.00 ± 5.189			
III-IV	15.00 ± 1.786			
**Tumor differentiation**		5.131	3.866–6.812	< 0.001
High and moderate	48.00 ± 3.288			
poor	9.00 ± 1.333			
**Tumor HDAC7 expression**		2.248	1.735–2.912	< 0.001
Low	41.00 ± 4.473			
High	13.00 ± 2.220			
**Tumor FGF18 expression**		2.029	1.578–2.608	< 0.001
Low	42.00 ± 4.321			
High	15.00 ± 2.620

**Table 3:** Multivariate analysis of the correlation between clinicopathological variables of NSCLC patients

Clinicopathological variables	Categories	Multivariate analysis		
		**HR**	**95% CI**	*P* **value**
TNM stages	I-II or III-IV	0.490	0.371–0.646	< 0.001
Tumor differentiation	High and moderate or poor	0.240	0.179–0.321	< 0.001
Tumor HDAC7 expression	Low or high	0.671	0.501–0.898	0.007
Tumor FGF18 expression	Low or high	0.720	0.539–0.961	0.026

### HDAC7 overexpression promotes NSCLC malignant progression

To investigate the role of HDAC7 on NSCLC malignant progression, we established HDAC7 overexpression and knockdown NSCLC cell lines with lentiviral infection based on the basal HDAC7 levels of NSCLC cell lines (Fig. S1a). Compared with control groups, HDAC7 overexpression remarkably increased cell viability detected by CCK-8 analysis (Fig. 1f). Moreover, EdU incorporation assay also showed that HDAC7 overexpression significantly increased the EdU-positive cells (Fig. 1g) indicating an increased cell proliferation. An increased clonogenic ability with HDAC7 overexpression was demonstrated in colony formation assay (Fig. 1h). We further measured the cell cycle–related proteins by western blotting and found that overexpression of HDAC7 dramatically upregulated CDK2 and Cyclin A2, while knockdown of HDAC7 downregulated these proteins (Fig. 1i). However, other cell cycle–related proteins did not significantly change in the cell lines, including CDK1, CDK4, and Cyclin D1 and so on. Additionally, Cyclin B1 was upregulated in HDAC7 overexpression cells, while did not change when HDAC7 was knocked down (Fig. S1b). Moreover, A549 shHDAC7 cells were synchronized at the G0/G1 by serum starvation, followed by the re-addition of serum (10% FBS) to allow cells to re-enter the cell cycle (Fig. S1c). We monitored HDAC7 and several cell cycle–related proteins at different time points. Interestingly, HDAC7 varied over time, and there was a HDAC7 expression peak in the control group. However, HDAC7 expression continuously declined in the shHDAC7 group. More importantly, the expression levels and trends of CDK2 and cyclin A2 were consistent with those of HDAC7 (Fig. 1j). To further verify the function of HDAC7 in promoting NSCLC cell proliferation in vivo, we performed xenograft assays on nude mice. Consistent with the in vitro results, the mean weight of subcutaneous tumors of the shHDAC7 group was significantly less than that in the control group (Fig. 1k). Additionally, mean tumors volume of HDAC7 overexpression group was significantly larger than that of control group (Fig. S1d).

**Fig. 2:**
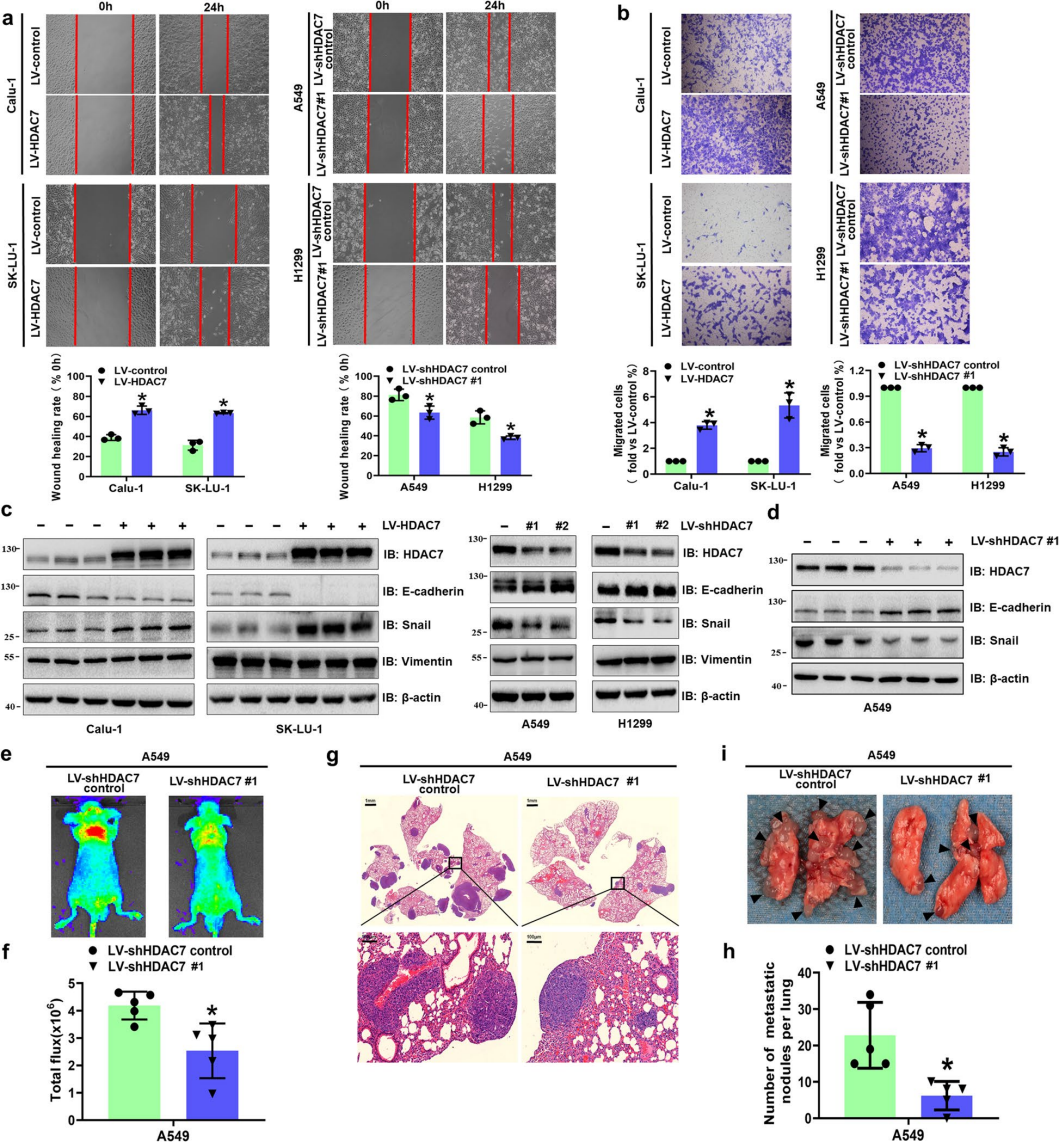
HDAC7 overexpression facilitated NSCLC metastasis in vitro and vivo. **a** Representative scratch wound assay images and results. Migration ability is expressed as the mean scratch area at each time point. The initial scratched area (0 h) was set as 100%. **b** Representative transwell migration assay images and results. Representative western blotting of HDAC7, E-cadherin, and Snail in HDAC7 overexpression and knockdown cells (**c**) and xenograft tumor tissues (**d**). Representative fluorescence images (**e**) and fluorescence signals analysis (**f**) of pulmonary metastases 4 weeks after tail vein injection. **g** Representative HE staining images of lung samples from indicated groups. Scale bar, 1 mm and 100 μm (inset), respectively. **h** The number of metastatic nodules per lung in HE staining images. **i** Representative general morphology of surface lung metastases. The black triangular arrows indicate the metastatic nodules. β-actin was used as internal control. All the data are expressed as mean ± SD. *P < 0.05. LV, lentivirus

The metastasizing capacities were also modulated by regulating HDAC7 expression. HDAC7 overexpression remarkably enhanced cell migration verified by scratch wound assay and transwell assay (Fig. 2a and b) and suggested that HDAC7 overexpression promoted the epithelial–mesenchymal transition (EMT) of the cells. We then analyzed the main EMT biomarkers by western blotting and found that HDAC7 overexpression significantly increased pro-EMT Snail levels and decreased anti-EMT E-cadherin levels, whereas the opposite trend was observed in the HDAC7 knockdown group both in vitro and in vivo (Fig. 2c and d). However, other EMT biomarkers, such as vimentin and ZEB1, did not change according to HDAC7 regulation (Fig. 2c, Fig. S1e). In vivo, the tail vein metastasis experiment showed that the downregulation of HDAC7 significantly lowered the incidence of lung metastasis and reduced the number of metastatic lung nodules (Fig. 2e–i). However, HDAC7 overexpression resulted in the opposite results (Fig. S1f-j).

### HDAC7 regulates cell proliferation and metastasis via FGF18 in NSCLC

**Fig. 3:**
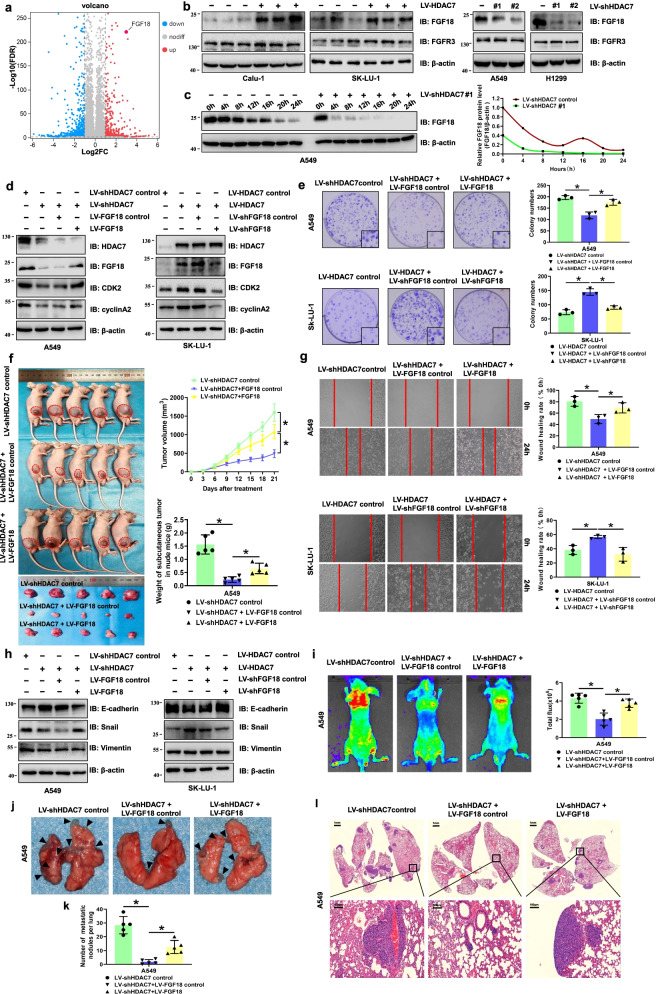
HDAC7 regulated cell proliferation and metastasis via FGF18 in NSCLC. **a** The volcano plot of DEGs identified from RNA-seq data of SK-LU-1 cells overexpressing HDAC7 or the control. **b** Representative western blotting of FGF18 and FGFR3 in HDAC7 overexpression and knockdown cells. **c** Representative western blotting of whole cell lysates (WCL) at indicated times. **d** Representative western blotting of HDAC7, FGF18, CDK2, and Cyclin A2 in the indicated groups. **e** Representative images and results of colony formation assay. **f** Gross photograph of subcutaneous xenograft tumors, tumor growth curve and tumor weight in indicated groups. **g** Representative scratch wound assay images and results. **h** Representative western blotting of E-cadherin, Snail, and vimentin in indicated groups. **i** Representative fluorescence images and data analysis of pulmonary metastases 4 weeks after tail vein injection. **j** The general morphology of surface lung metastases. The black triangular arrows indicate the metastatic nodules. The number of metastatic nodules per lung in HE images (**k**) and representative HE staining images (**l**) from lung samples of indicated groups. Scale bar, 1 mm and 100 μm (inset), respectively. β-actin was used as internal control. All the data are expressed as mean ± SD. *P < 0.05. LV, lentivirus

To explore the potential mechanisms of HDAC7-enhanced NSCLC progression, the RNA-seq based transcriptome analysis was performed in SK-LU-1 cells to compare transcriptome changes between control and HDAC7 overexpression (OE) groups. In total, there were 432 genes upregulated and 654 genes downregulated in the HDAC7 OE group compared with control group. The up- or downregulated genes with > twofold differential expression are shown in the heatmap (Fig. S2a). The GO function enrichment analysis indicated that DEGs were closely related to cell growth, motility and differentiation, etc. (Fig. S2b). Among these genes, we found that a set of FGF family genes, including FGF9, FGF14 and FGF18 were upregulated by HDAC7 overexpression. However, the volcano plot showed that FGF18 is one of the top-ranked differentially expressed genes (Fig. 3a). Previous studies suggested that the amplification of FGF18 was found in a number of solid tumors, and its overexpression was significantly correlated with poor prognosis [21–24]. Considering the important roles of both FGF18 and HDAC7 in NSCLC progression, we were interested in investigating their regulatory relationship and underlying mechanisms. Western blotting validated that the FGF18 protein levels were significantly upregulated in HDAC7 overexpressing cells. Furthermore, knockdown of HDAC7 could significantly downregulate FGF18 expression (Fig. 3b). In addition, the feature of FGF18 expression was closely consistent with that of HDAC7 expression (Fig. 3c). Moreover, our preliminary data showed FGF18 in culture medium released from NSCLC cells was also increased after overexpressing HDAC7 (Fig. S2c).

To further confirm the involvement of FGF18 in HDAC7-regulated NSCLC progression, we upregulated FGF18 in A549 HDAC7 knockdown cells and downregulated FGF18 in SK-LU-1 HDAC7 overexpressing cells using lentivirus infection. Western blotting showed that overexpressing or knockdown FGF18 had no effect on HDAC7 protein levels of these cells. However, FGF18 overexpression or knockdown could partially reverse the HDAC7-induced cell cycle–related protein changes (Fig. 3d). The colony formation assay showed that FGF18 knockdown partially rescued the enhanced proliferative ability of HDAC7 overexpressing cells (Fig. 3e). Furthermore, the in vivo assay indicated that FGF18 overexpression significantly increased subcutaneous tumor weight in the A549 shHDAC7 group (Fig. 3f). Moreover, FGF18 silencing could dramatically attenuate subcutaneous tumor growth in the Calu-1 HDAC7 overexpression group (Fig. S2d).

Scratch wound assay revealed that FGF18 overexpression significantly enhanced the migratory abilities of A549 shHDAC7 cells, whereas HDAC7-enhanced migratory abilities were rescued by FGF18 knockdown (Fig. 3g). Western blotting indicated that FGF18 knockdown reversed the HDAC7 overexpression–induced EMT marker changes by increasing E-cadherin and decreasing Snail protein levels in vitro (Fig. 3h). Furthermore, tail vein metastasis assay showed the nude mice injected with FGF18-overexpressing cells presented an enhanced incidence of lung metastasis, a dramatically increased number of lung metastatic nodules, and more intense fluorescence imaging signals than did the A549 shHDAC7 group (Fig. 3i–l). However, silencing FGF18 in the Calu-1 overexpression cells showed the opposite results (Fig. S2e-i). Taken together, the results suggest that FGF18 is essential for HDAC7-mediated NSCLC proliferation and metastasis.

### HDAC7 is involved in the deacetylation of β-catenin and promotes its translocation into the nucleus to activate FGF18 expression

**Fig. 4:**
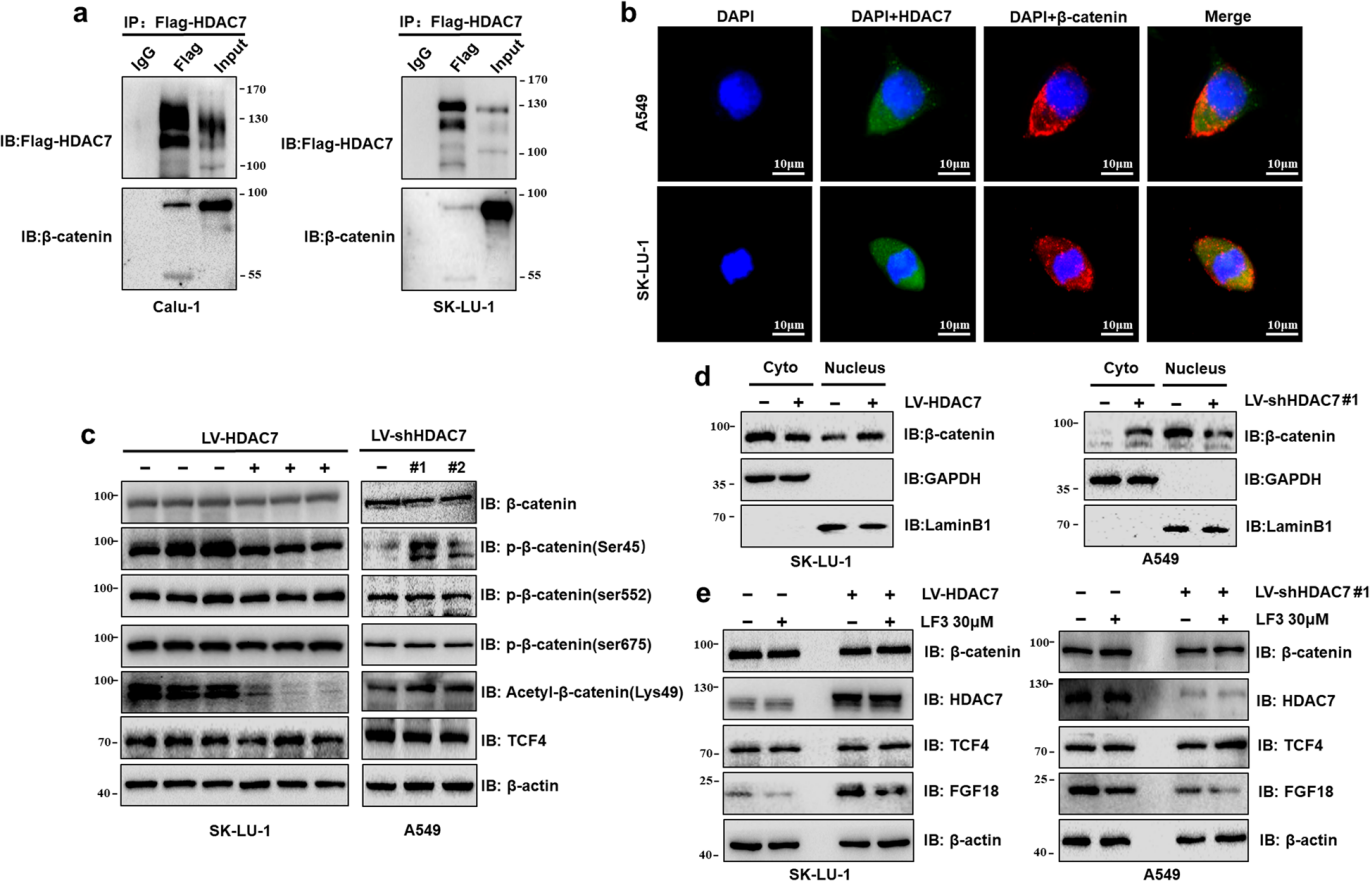
HDAC7 deacetylated β-catenin at Lys49 and promoted its translocation into the nucleus to activate FGF18 expression. **a** HDAC7 and β-catenin interacted in NSCLC cells. **b** Two-color immunofluorescence analysis showing significant colocalization of HDAC7 with β-catenin in A549 and SK-LU-1 cells. DAPI (blue), HDAC7 (green), and β-catenin (red). **c** Representative western blotting results showing HDAC7 deacetylated β-catenin at Lys49 and phosphorylated β-catenin at Ser45. **d** Representative western blotting showing upregulated HDAC7 facilitated β-catenin nuclear translocation. **e** Representative western blotting of HDAC7, FGF18, β-catenin, and TCF4 in NSCLC cells after LF3 treatment. β-actin was used as an internal control to normalize the total protein amount. GAPDH and Lamin B1 were used as the loading control to cytosolic protein and nuclear protein, respectively. DAPI fluorescence marking the cell nucleus. LV, lentivirus. IP, immunoprecipitation. IB, immunoblot

Given that the activation of β-catenin pathway has been reported in a significant proportion of NSCLC cases by other mechanisms, we predicted whether HDAC7 regulates FGF18 expression via the β-catenin pathway. Coimmunoprecipitation confirmed an interaction between HDAC7 and β-catenin in NSCLC cells (Fig. 4a). The immunofluorescence staining revealed the subcellular colocalization of HDAC7 (green) and β-catenin (red) in NSCLC cells (Fig. 4b). The above results suggested the possibility that β-catenin might be a substrate of HDAC7. According to the previous studies reporting that HDAC6 could deacetylate β-catenin at Lys49 [25], we further investigated whether HDAC7 had a similar effect on NSCLC. As shown in Fig. 4c, the total level of β-catenin did not change after HDAC7 was regulated and TCF4, a main nuclear effector of β-catenin signaling on FGF18 [26], did not change either. However, the acetylation of β-catenin at Lys49 was significantly decreased in HDAC7 overexpressing cells and conversely, the level of acetyl-β-catenin (Lys49) was significantly increased when HDAC7 was downregulated. We also monitored several common phosphorylation sites of β-catenin. Interestingly, the phosphorylation level of β-catenin at Ser45, but not at Ser552 or Ser675, was significantly decreased in HDAC7 upregulated cells. The nuclear-cytosolic protein isolation assay also revealed that HDAC7 overexpression promoted β-catenin nuclear import (Fig. 4d). To further confirm that transcriptional activation of FGF18 is directly affected by the β-catenin/TCF4 complex, LF3, a robust specific antagonist of the β-catenin/TCF4 interaction, was used in our experiment. As shown in Fig. 4e, LF3 did not affect the protein levels of β-catenin, TCF4, or HDAC7. However, LF3 could significantly decrease the expression level of FGF18. TOP/FOP flash activity assay additionally indicated that HDAC7 overexpression significantly activated β-catenin signaling, while HDAC7 knockdown dramatically suppressed it, confirming that β-catenin signaling is a downstream target activated by HDAC7 in NSCLC cells (Fig. S3a). Moreover, HDAC7 overexpression in SK-LU-1 cells significantly upregulated c-Myc and MMP7 both at mRNA and protein levels. In contrast, knocking down HDAC7 in A549 cells diminished the expression of β-catenin related genes (Fig. S3b and c). Lastly, HDAC7 silencing was performed in both SK-LU-1 and Calu-1 HDAC7 overexpression cells. As shown in Fig. S3d, the data further validated the pathway involved did not result from off-target or other effects (Fig. S3d).

### USP10 interacts with and stabilizes HDAC7

**Fig. 5:**
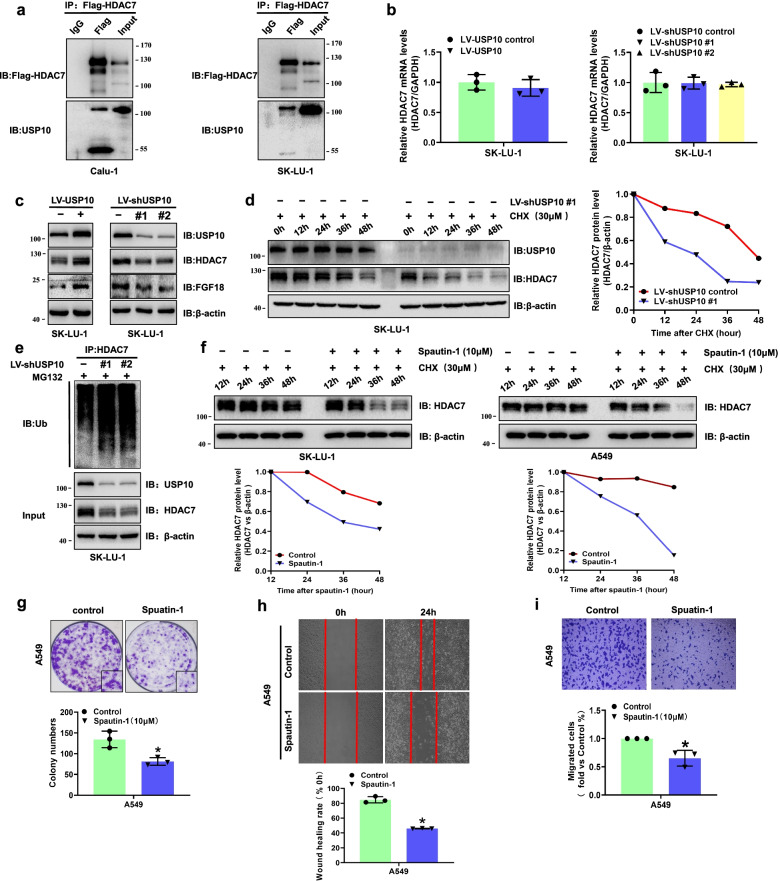
USP10 interacted with and stabilized HDAC7. **a** USP10 and HDAC7 interacted in NSCLC cells. **b** The statistical analysis of relative HDAC7 mRNA levels in USP10 overexpression and knockdown cells. mRNA was normalized to GAPDH. **c** Representative western blotting of USP10, HDAC7 and FGF18 in indicated cells. **d** Protein stability analysis showing a reduced half-life of HDAC7 in USP10 knockdown cells. **e** Knockdown of USP10 promoted ubiquitination of HDAC7 in NSCLC cells. IP with anti-HDAC7 antibody was followed by IB with anti-ubiquitin antibody to detect polyubiquitinated HDAC7. **f** Representative western blotting of USP10 inhibitor indicating that Spautin-1 reduced the half-life of HDAC7. The NSCLC cells were treated with Spautin-1 at the indicated concentration and time intervals. For graphs in d and f, the relative HDAC7 levels were quantified using Image Lab software. Representative images and statistical analysis of colony formation assay (**g**), scratch wound assay (**h**), and transwell assay (**i**) in A549 cells after treatment with Spautin-1. Migration ability is expressed as the mean scratch area at each time point. The initial scratched area (0 h) was set as 100%. β-actin was used as the internal control. LV, lentivirus. IP, immunoprecipitation. IB, immunoblot

We then explored whether the stability of HDAC7 protein is affected by ubiquitination. Firstly, mass spectrometry analysis of recombinant HDAC7 (Flag-HDAC7) in proliferating 293 T cells was performed. USP10 was identified as an original HDAC7-binding protein (Fig. S3e). To verify this interaction, we further immunoprecipitated Flag-HDAC7 and USP10 in HDAC7 overexpressing cells. As shown in Fig. 5a, the anti-USP10 antibody, but not the anti-IgG antibody, immunoprecipitated HDAC7. Next, we established USP10 overexpression or knockdown NSCLC cell lines with lentiviral infection. The qRT-PCR showed HDAC7 mRNA levels were not affected by the change of USP10 (Fig. 5b). As shown in Fig. 5c, overexpressing USP10 could significantly increase HDAC7 protein levels. We also observed a significant decrease of HDAC7 expression in USP10 knockdown cells. Based on the above results, USP10 may most likely regulate HDAC7 through deubiquitinating HDAC7 and thus stabilizing it. To further confirm our hypothesis, we monitored the decay rate of HDAC7 by treating indicated cells with cycloheximide (CHX). HDAC7 protein showed faster degradation in USP10 knockdown cells than that in the control group (Fig. 5d). Moreover, knockdown of USP10 dramatically increased the ubiquitination of HDAC7 (Fig. 5e). Spautin-1, the specific inhibitor of USP10, could also accelerate the degradation of HDAC7 in both A549 and SK-LU-1 cells (Fig. 5f). Furthermore, the inhibition of USP10 could significantly attenuate the proliferation and motility of A549 cells (Fig. 5g–i). In order to investigate whether USP10 and HDAC7 signaling is specific for NSCLC tissues, we further analyzed the USP10 and HDAC7 co-expression by IHC staining in 71 paired tumor–normal tissue samples (Fig. S3f). Interestingly, we found that there is a significant correlation between USP10 and HDAC7 expression in NSCLC tissues (*p* < 0.001, Fig. S3g) but not in paired normal tissue (*p* = 0.254, Fig. S3h). Taken together, our preliminary results revealed that HDAC7 may be the potential USP10 substrate in NSCLC and inhibition of USP10 could reduce the deubiquitination of HDAC7 and weaken progression of NSCLC cells.

### The correlation of HDAC7 and FGF18 expression with NSCLC prognosis

**Fig. 6:**
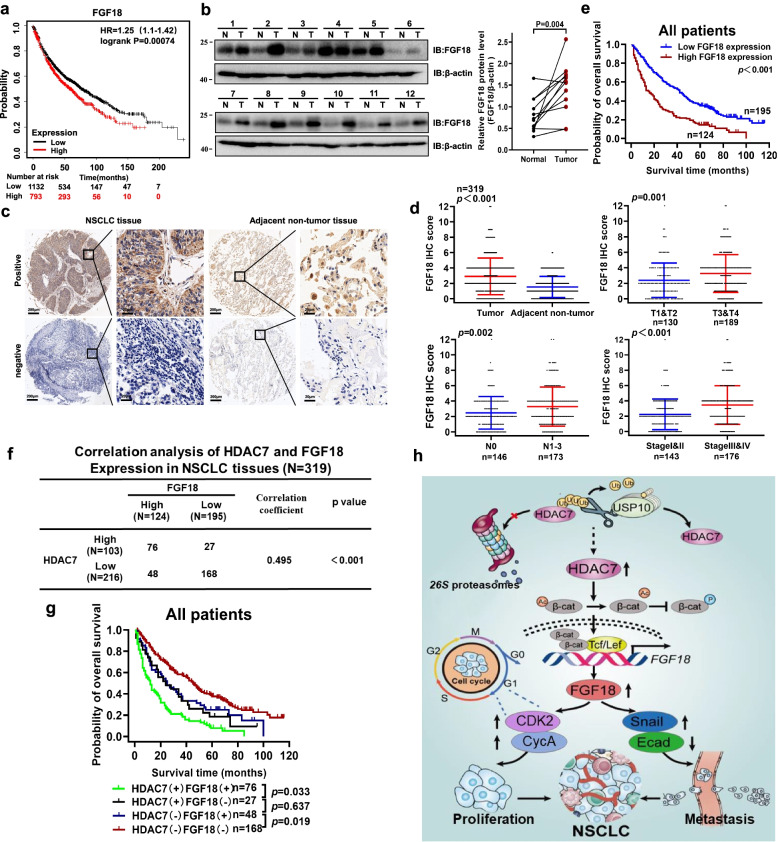
The correlation of HDAC7 and FGF18 expression in NSCLC. **a** Kaplan–Meier survival analysis of FGF18 expression in 1925 NSCLC cases from the Kaplan–Meier plotter database. **b** Western blot analysis of FGF18 expression in tumor (T) and paired adjacent nontumor (N) tissues from 12 NSCLC patients. (**c**) Representative FGF18 IHC images of NSCLC and adjacent non-tumor tissues. Scale bar, 200 μm and 20 μm (inset), respectively. **d** The IHC analysis of FGF18 expression in 319 NSCLC patients. **e** Kaplan–Meier survival analysis of FGF18 expression in 319 NSCLC patients. **f** Correlation analysis of HDAC7 and FGF18 expression in NSCLC tissues. **g** The comprehensive Kaplan–Meier survival analysis of HDAC7 and FGF18 in 319 NSCLC patients based on the microarray tissue IHC results. **h** The schematic of a simplified regulatory network involving USP10 and FGF18 in HDAC7-meditated NSCLC progression. USP10 interacts with and stabilizes HDAC7 through deubiquitination, preventing its proteasomal degradation. Elevated HDAC7 expression facilitates β-catenin redistribution to the nucleus by deacetylating β-catenin and inhibiting its phosphorylation. As a result, the nuclear accumulation of β-catenin binds to TCF/LEF to activate the transcription of FGF18 and thus promotes NSCLC proliferation and metastasis

The data from the Kaplan–Meier plotter database showed that high expression of FGF18 was closely correlated with poor prognosis of NSCLC patients (Fig. 6a). Though FGF18 levels of 1/12 patients did not change, statistical analysis indicated the FGF18 levels in the tumors to be significantly higher than in normal tissues (*P* = 0.004, Fig. 6b). We further investigated FGF18 expression by IHC tissue array analysis (Fig. 6c). The FGF18 expression in tumors was significantly higher than that in adjacent nontumor tissues (Fig. 6d). In addition, the NSCLC patients with deep tumor invasion and/or larger tumors (T3–T4), high stages (stage III–IV), and lymph node metastasis (N1–N3) had higher FGF18 expression (Fig. 6d). The NSCLC patients with elevated FGF18 expression showed both shortened overall and median survival times (Fig. 6e, Table 2). The elevated FGF18 expression was also positively correlated to larger tumor size, T stage, lymphatic invasion, distant metastasis, TNM stages, and tumor differentiation (Table 1). Correlation analysis showed a significant correlation between HDAC7 and FGF18 expression in NSCLC tissues (Fig. 6f). Moreover, Cox regression analyses showed that FGF18 was an independent prognostic factor for NSCLC patients (Table 3). Furthermore, we found that HDAC7/FGF18 co-expression was significantly correlated with the poorest prognosis in a subset of NSCLC patients. Meanwhile, the patients with low HDAC7 and FGF18 expression had the longest overall survival time (Fig. 6g). Overall, HDAC7 expression was positively correlated with FGF18 expression, and NSCLC patients with co-expression of HDAC7 and FGF18 demonstrated the poorest prognosis.

## Discussion

NSCLC is the most common histopathological type of lung cancer, which is the leading cause of cancer death worldwide [1]. The molecular mechanisms underlying NSCLC progression require further clarification for the development of more effective therapeutic approaches. In this study, we indicated that HDAC7 could promote NSCLC progression via posttranslational modification of β-catenin, thereby facilitating its nuclear transfer and activating the downstream target FGF18. In addition, our preliminary results demonstrated that deubiquitinase USP10 acts as a regulator of HDAC7, which deubiquitinates and stabilizes HDAC7 to prevent its degradation in NSCLC, thus representing a potential novel therapeutic target.

HDAC7 is a key factor in the regulation of cellular processes, such as proliferation, migration, and apoptosis [11]. Several previous studies have demonstrated that HDAC7 has a carcinogenic role in breast cancer, ovarian cancer, and glioma [13–15]. Moreover, overexpression of HDAC7 is correlated with poor prognosis in gastric cancer and hematological malignancies [12, 16]. Recently, Lei et al. evaluated the data from 484 lung cancer samples from The Cancer Genome Atlas (TCGA), and claimed that high HDAC7 mRNA level was correlated with poor prognosis of lung cancer patients [19]. In the present study, the TMA analysis showed HDAC7 expression was significantly upregulated in NSCLC tissues and closely correlated with poor NSCLC prognosis. Moreover, as an independent prognostic factor for NSCLC patients, high HDAC7 was positively correlated with TNM stage and tumor differentiation. These results further strengthened the oncogenic role of HDAC7 in NSCLC.

Previous studies have found that HDAC7 is a crucial pro-proliferative factor of cancer cells, such as Hela, HCT116, and MCF-7 [27, 28]. Recent studies on lung cancer have also shown that HDAC7 has positive effects on tumorigenesis and cell growth via inhibition of Stat3 and plakoglobin [18, 19]. In our study, we found that overexpression of HDAC7 remarkably enhanced the proliferative abilities of NSCLC cells both in vitro and in vivo. These findings provide some degree of support to the notion that patients with upregulated HDAC7 have larger maximal tumor size and higher clinical T stage. Moreover, we observed positive effects of HDAC7 on cell cycle regulatory molecules, which indicated that HDAC7-mediated NSCLC proliferation may be most likely due to facilitating the bypass of S phase and G2/M phases. The results of the cell cycle analysis were in line with these conclusions.

Previous studies have also shown the critical role of HDAC7 in cancer metastasis, *e.g.* in nasopharyngeal [29], breast, and ovarian cancer [14]. Similar to a previous study by Sang et al [18], we observed an increased migration capacity after upregulating HDAC7 in NSCLC cells. This further supported our previous IHC results that NSCLC with high expression of HDAC7 exhibited advanced lymph node and distant metastasis. As EMT is considered to be vital process in cancer metastasis, we further investigated whether there is any relationship between HDAC7-induced metastasis and EMT-related molecules. The subsequent analysis confirmed this conjecture, which indicated NSCLC metastasis may have arisen from HDAC7-mediated variation of specific EMT biomarker expressions.

Although we verified the function of HDAC7 in aggravating NSCLC proliferation and metastasis, the underlying mechanisms driving tumor progression need to be further elaborated. In the present study, we focused on FGF18, which is involved in HDAC7-mediated NSCLC progression. Previous studies have reported a prominent role of FGF18 in tumorigenesis, proliferation, and metastasis of several tumors, including gastric cancer, ovarian cancer, and breast cancer [21, 22, 24]. Chen et al. found that the FGF18 could promote proliferation and migration of large cell lung carcinoma (LCLC) cells [30]. Unfortunately, clinical data concerning the roles of FGF18 in NSCLC are insufficient for determining their exact function in NSCLC. In the current study, IHC staining in TMA showed high FGF18 expression in NSCLC was not only associated with advanced clinical stage and poorer prognosis of NSCLC patients, but also positively correlated with HDAC7 expression. The mechanistic studies further demonstrated that HDAC7 positively regulates the expression of FGF18. Remarkably, both in vitro and in vivo experiments confirmed that FGF18 knockdown could abrogate HDAC7-induced NSCLC proliferation and metastasis. These results indicated that FGF18 is a crucial downstream oncogenic protein of HDAC7, and it may be closely involved in HDAC7-mediated NSCLC progression.

Further explorations are necessary to clarify the potential mechanisms by which HDAC7 regulates FGF18 expression in NSCLC. β-catenin is the key mediator of the Wnt/β-catenin signaling pathway. Aberrant Wnt/β-catenin signaling is causatively associated with a wide variety of diseases, including cancer [31, 32]. One previous study showed that targeted disruption of β-catenin significantly blocked FGF18 expression [33], while another found that β-catenin is capable of enhancing FGF18 transcription in colon cancer from the promoter of FGF18 harboring TCF4-binding motifs [26]. These studies demonstrate that FGF18 is a vital downstream transcription target of β-catenin pathway. Interestingly, a recent finding in glioma indicated that decreased acetylation levels of β-catenin induced by HDAC7 changed steric hindrance of β-catenin, thus inhibiting its phosphorylation level and activation of the Wnt pathway [15]. This is also consistent with previous research on HDAC6 that reported β-catenin to be a nonhistone substrate regulated by HDAC modification [25]. Here, we focused on the effects of HDAC7 on β-catenin regulation in NSCLC. Our data showed that HDAC7 interacted with and largely co-localized with β-catenin in NSCLC cells. Despite levels of total β-catenin did not significantly changed after regulation of HDAC7, we observed upregulated HDAC7 dramatically decreased β-catenin acetylation at Lys49 and phosphorylation at Ser45, which facilitated translocation of β-catenin from cytoplasm to nucleus. Subsequently, it triggered FGF18 expression via β-catenin–TCF4 complex, which further underpins the results reported previously [26]. Therefore, we here report a HDAC7-β-catenin–FGF18 regulatory axis, which is involved in the malignant progression of NSCLC.

Several previous evidence have revealed the complex roles of USP10 in malignant progression of multiple cancers. Although USP10 could inhibit cell growth via stabilization of p53 in cancer cells with wild-type p53, it could also promote cancer cell proliferation in mutant p53 background [34]. Moreover, USP10 could inhibit c-Myc transcriptional activation via stabilization of SIRT6 to inhibit tumor formation in colon cancer [35]. In contrast, USP10 promotes proliferation and metastasis of hepatocellular carcinoma by targeting YAP/TAZ and Smad4, respectively [36, 37]. Weisberg et al. found that USP10 deubiquitinates and stabilizes oncogenic FLT3 to promote tumor progression [38]. These studies indicated that the complicated functions of USP10 are largely dependent on its deubiquitinated substrates in the specific cell context of cancer types. However, the research regarding USP10 in lung cancer is still limited, and available results are also controversial. A recent study reported that USP10 regulates KLF4 stability and exerts a tumor-suppressive role in lung cancer [39]. On the contrary, Hu et al. found that USP10 plays a growth-promoting role in NSCLC via stabilizes HDAC6 [40]. Similar to the reported by Hu et al., we discovered that USP10 deubiquitinates and stabilizes HDAC7 in vivo, which in turn promotes growth and migration of NSCLC cells. In addition, a specific co-expression of USP10 and HDAC7 were observed in NSCLC tissues but not in normal. Thus, further in-depth experimental and more clinical data are necessary to elucidate the clinical relevance and mechanisms of USP10 and HDAC7 in this disease context.

Despite these interesting findings, several limitations should also be acknowledged. One limitation is that since the key problem in our current research was centered around the role and mechanism of HDAC7, downstream signaling of FGF18 has not been thoroughly investigated here. In addition, whether HDAC7 has other effects on β-catenin in three-dimensional structure alterations, conformation changes, potential disruption or creation of other protein–protein interaction surfaces and so on remains unclear. Therefore, our future experiments are targeted to answer these important questions.

## Conclusion

We found a novel HDAC7-β-catenin–FGF18 pathway that was involved in NSCLC proliferation and metastasis. Our preliminary data also provide insights into the function of USP10 in NSCLC cells suggesting innovative strategies for future antitumor drug development and drug resistance research.

## Supplementary Information

**Additional file 1: Figure 1. (a) **The basal HDAC7 expressions of NSCLC cell lines.** (b)** Representative western blotting of cell cycle–related proteins in HDAC7 overexpression and knockdown cells. **(c) **Flow cytometry results of cell-cycle distribution at indicated time points. **(d) **Gross photograph of subcutaneous xenograft tumors, tumor growth curve and subcutaneous tumor weight in each group. **(e)** Representative western blot of EMT-associated proteins in HDAC7 overexpression and knockdown cells. Representative fluorescence images **(f)** and fluorescence signals analysis **(g)** of pulmonary metastases 4 weeks after tail vein injection. **(h)** Representative HE staining images of lung samples from indicated groups. Scale bar, 1 mm and 100 μm (inset), respectively. (**i)** The number of metastatic nodules per lung in HE staining images.** (j)** Representative general morphology of surface lung metastases. The black triangular arrows indicate the metastatic nodules. β-actin was used as internal control. All the data are expressed as mean ± SD. *P < 0.05. LV, lentivirus.

**Additional file 2: Figure 2. (a)** Heatmap of RNA-seq data from SK-LU-1 cells overexpressing HDAC7 or the control based on the log2 intensity. **(b) **The GO function enrichment analysis of DEGs in SK-LU-1 cells overexpressing HDAC7 or the control, ranked by the P value. **(c) **The quantification changes of FGF18 in the culture medium after up- or downregulating HDAC7 in NSCLC cells. **(d)** Gross photograph of subcutaneous xenograft tumors, tumor growth curve and tumor weight in indicated groups. Representative fluorescence images **(**e**)** and fluorescence signals analysis **(**f**)** of pulmonary metastases 4 weeks after tail vein injection. **(**g**) **Representative HE staining images of lung samples from indicated groups. Scale bar, 1 mm and 100 μm (inset), respectively. **(**h**)** The number of metastatic nodules per lung in HE staining images. **(**i**)** Representative general morphology of surface lung metastases. The black triangular arrows indicate the metastatic nodules. All the data are expressed as mean ± SD. *P < 0.05. LV, lentivirus.

**Additional file 3: Figure 3. (a) **The relative TOP/FOP ratio in HDAC7 overexpression or knockdown NSCLC cells. The mRNA **(b) **and protein levels** (c) **of β-catenin related genes in HDAC7 overexpression or knockdown NSCLC cells. **(d) **Representative western blotting of HDAC7-β-catenin-FGF18 pathway involved proteins in indicated NSCLC cells. **(e) **Mass spectrometry analysis results of USP10 peptide sequence (IAELLENVTLIHKPVSLQP). **(f)** Representative HDAC7 and USP10 IHC images of NSCLC and adjacent non-tumor tissues. Scale bar, 200 μm and 50 μm (inset), respectively. Correlation analysis of HDAC7 and USP10 expression in NSCLC tissues **(g)** and in adjacent non-tumor tissues** (h)**. β-actin was used as internal control. All the data are expressed as mean ± SD. *P < 0.05. LV, lentivirus.

**Additional file 4.**

## Data Availability

The datasets used and analyzed during the current study are available from the corresponding author on reasonable request.

## Abbreviations

HDAC7: Histone deacetylases 7
FGF 18: Fibroblast growth factor 18
FGFRs: Fibroblast growth factor receptors
LRP5/6: Lipoprotein receptor-related protein 5/6
GSK-3β: Glycogen synthase kinase-3β
CK1: Casein kinase 1
APC: Adenomatous polyposis coli
TCF: T cell specific factor
LEF: Lymphoid enhancer-binding factor
USP10: Ubiquitin-specific peptidase 10
TMA: Tissue microarray

## References

[1] Bray F, Ferlay J, Soerjomataram I, Siegel RL, Torre LA, Jemal A (2018). Global cancer statistics 2018: GLOBOCAN estimates of incidence and mortality worldwide for 36 cancers in 185 countries. CA Cancer J Clin.

[2] Li G, Tian Y, Zhu WG (2020). The Roles of Histone Deacetylases and Their Inhibitors in Cancer Therapy. Front Cell Dev Biol..

[3] Asfaha Y (2019). Recent advances in class IIa histone deacetylases research. Bioorg Med Chem..

[4] Wei JY, Li WM, Zhou LL, Lu QN, He W (2015). Melatonin induces apoptosis of colorectal cancer cells through HDAC4 nuclear import mediated by CaMKII inactivation. J Pineal Res.

[5] Kaowinn S (2017). Increased EGFR expression induced by a novel oncogene, CUG2, confers resistance to doxorubicin through Stat1-HDAC4 signaling. Cell Oncol (Dordr).

[6] Amodio N (2016). Therapeutic Targeting of miR-29b/HDAC4 Epigenetic Loop in Multiple Myeloma. Mol Cancer Ther.

[7] Huang Y, Tan M, Gosink M, Wang KK, Sun Y (2002). Histone deacetylase 5 is not a p53 target gene, but its overexpression inhibits tumor cell growth and induces apoptosis. Cancer res.

[8] Huang Y, Jian W, Zhao J, Wang G (2018). Overexpression of HDAC9 is associated with poor prognosis and tumor progression of breast cancer in Chinese females. Onco Targets Ther.

[9] Zhang Y (2016). Downregulation of HDAC9 inhibits cell proliferation and tumor formation by inducing cell cycle arrest in retinoblastoma. Biochem Biophys Res Commun.

[10] Ma Z (2019). Histone deacetylase 9 downregulation decreases tumor growth and promotes apoptosis in non-small cell lung cancer after melatonin treatment. J Pineal Res..

[11] Parra M (2015). Class IIa HDACs - new insights into their functions in physiology and pathology. FEBS J.

[12] Yu Y, Cao F, Yu X, Zhou P, Di Q, Lei J (2017). The expression of HDAC7 in cancerous gastric tissues is positively associated with distant metastasis and poor patient prognosis. Clin Transl Oncol.

[13] Wu MY, Fu J, Xiao X, Wu J, Wu RC (2014). MiR-34a regulates therapy resistance by targeting HDAC1 and HDAC7 in breast cancer. Cancer lett.

[14] Witt AE, Lee CW, Lee TI, Azzam DJ, Wang B, Caslini C (2017). Identification of a cancer stem cell-specific function for the histone deacetylases, HDAC1 and HDAC7, in breast and ovarian cancer. Oncogene.

[15] Yu X, Wang M, Wu J, Han Q, Zhang X (2019). ZNF326 promotes malignant phenotype of glioma by up-regulating HDAC7 expression and activating Wnt pathway. J Exp Clin Cancer Res.

[16] Moreno DA, Scrideli CA, Cortez MA, de Paula QR, Valera ET, da Silva SV (2010). Differential expression of HDAC3, HDAC7 and HDAC9 is associated with prognosis and survival in childhood acute lymphoblastic leukaemia. Br J Haematol.

[17] Barneda-Zahonero B, Collazo O, Azagra A, Fernández-Duran I, Serra-Musach J, Islam AB (2015). The transcriptional repressor HDAC7 promotes apoptosis and c-Myc downregulation in particular types of leukemia and lymphoma. Cell Death Dis..

[18] Sang Y, Sun L, Wu Y, Yuan W, Liu Y, Li S-W (2019). Histone deacetylase 7 inhibits plakoglobin expression to promote lung cancer cell growth and metastasis. Int J Oncol.

[19] Lei Y, Liu L, Zhang S, Guo S, Li X, Wang J (2017). Hdac7 promotes lung tumorigenesis by inhibiting Stat3 activation. Mol Cancer.

[20] Liu D, Ma Z, Di S, Yang Y, Yang J, Xu L (2018). AMPK/PGC1alpha activation by melatonin attenuates acute doxorubicin cardiotoxicity via alleviating mitochondrial oxidative damage and apoptosis. Free Radic Biol Med.

[21] Zhang J, Zhou Y, Huang T, Wu F, Pan Y, Dong Y (2018). FGF18, a prominent player in FGF signaling, promotes gastric tumorigenesis through autocrine manner and is negatively regulated by miR-590-5p. Oncogene.

[22] Wei W, Mok SC, Oliva E, Kim SH, Mohapatra G, Birrer MJ (2013). FGF18 as a prognostic and therapeutic biomarker in ovarian cancer. J Clin Invest.

[23] Sonvilla G, Allerstorfer S, Stättner S, Karner J, Klimpfinger M, Fischer H (2008). FGF18 in colorectal tumour cells: autocrine and paracrine effects. Carcinogenesis.

[24] Yu Z, Lou L, Zhao Y (2018). Fibroblast growth factor 18 promotes the growth, migration and invasion of MDA-MB-231 cells. Oncol Rep.

[25] Li Y, Zhang X, Polakiewicz RD, Yao TP, Comb MJ (2008). HDAC6 is required for epidermal growth factor-induced beta-catenin nuclear localization. J Biol Chem.

[26] Shimokawa T, Furukawa Y, Sakai M, Li M, Miwa N, Lin YM (2003). Involvement of the FGF18 gene in colorectal carcinogenesis, as a novel downstream target of the beta-catenin/T-cell factor complex. Cancer Res.

[27] Zhu C, Chen Q, Xie Z, Ai J, Tong L, Ding J (2011). The role of histone deacetylase 7 (HDAC7) in cancer cell proliferation: regulation on c-Myc. J Mol Med.

[28] Wang S, Li X, Parra M, Verdin E, Bassel-Duby R, Olson EN (2008). Control of endothelial cell proliferation and migration by VEGF signaling to histone deacetylase 7. Proc Natl Acad Sci U S A.

[29] Li QG, Xiao T, Zhu W, Yu ZZ, Huang XP, Yi H (2020). HDAC7 promotes the oncogenicity of nasopharyngeal carcinoma cells by miR-4465-EphA2 signaling axis. Cell Death Dis.

[30] Chen T, Gong W, Tian H, Wang H, Chu S, Ma J (2017). Fibroblast growth factor 18 promotes proliferation and migration of H460 cells via the ERK and p38 signaling pathways. Oncol Rep.

[31] Moon RT, Kohn AD, De Ferrari GV, Kaykas A (2004). WNT and beta-catenin signalling: diseases and therapies. Nat Rev Genet.

[32] Nusse R, Clevers H (2017). Wnt/β-Catenin Signaling, Disease, and Emerging Therapeutic Modalities. Cell.

[33] Reinhold MI, Naski MC (2007). Direct interactions of Runx2 and canonical Wnt signaling induce FGF18. J Biol Chem.

[34] Yuan J, Luo K, Zhang L, Cheville JC, Lou Z (2010). USP10 Regulates p53 Localization and Stability by Deubiquitinating p53. Cell.

[35] Lin Z, Yang H, Tan C, Li J, Liu Z, Quan Q (2013). USP10 antagonizes c-Myc transcriptional activation through SIRT6 stabilization to suppress tumor formation. Cell Rep.

[36] Zhu H, Yan F, Yuan T, Qian M, Zhou T, Dai X (2020). USP10 Promotes Proliferation of Hepatocellular Carcinoma by Deubiquitinating and Stabilizing YAP/TAZ. Cancer Res.

[37] Yuan T, Chen Z, Yan F, Qian M, Luo H, Ye S (2020). Deubiquitinating enzyme USP10 promotes hepatocellular carcinoma metastasis through deubiquitinating and stabilizing Smad4 protein. Mol Oncol.

[38] Weisberg EL, Schauer NJ, Yang J, Lamberto I, Doherty L, Bhatt S (2017). Inhibition of USP10 induces degradation of oncogenic FLT3. Nat Chem Biol.

[39] Wang X, Xia S, Li H, Wang X, Li C, Chao Y (2020). The deubiquitinase USP10 regulates KLF4 stability and suppresses lung tumorigenesis. Cell Death Differ.

[40] Hu C, Zhang M, Moses N, Hu CL, Polin L, Chen W (2020). The USP10-HDAC6 axis confers cisplatin resistance in non-small cell lung cancer lacking wild-type p53. Cell Death Dis.

