# Supplementary figures and images for: HDAC7 promotes NSCLC proliferation and metastasis via stabilization by deubiquitinase USP10 and activation of β-catenin-FGF18 pathway
Source: J Exp Clin Cancer Res. 2022 Mar 11;41:91. doi: 10.1186/s13046-022-02266-9 (PMC8915541; doi:10.1186/s13046-022-02266-9)

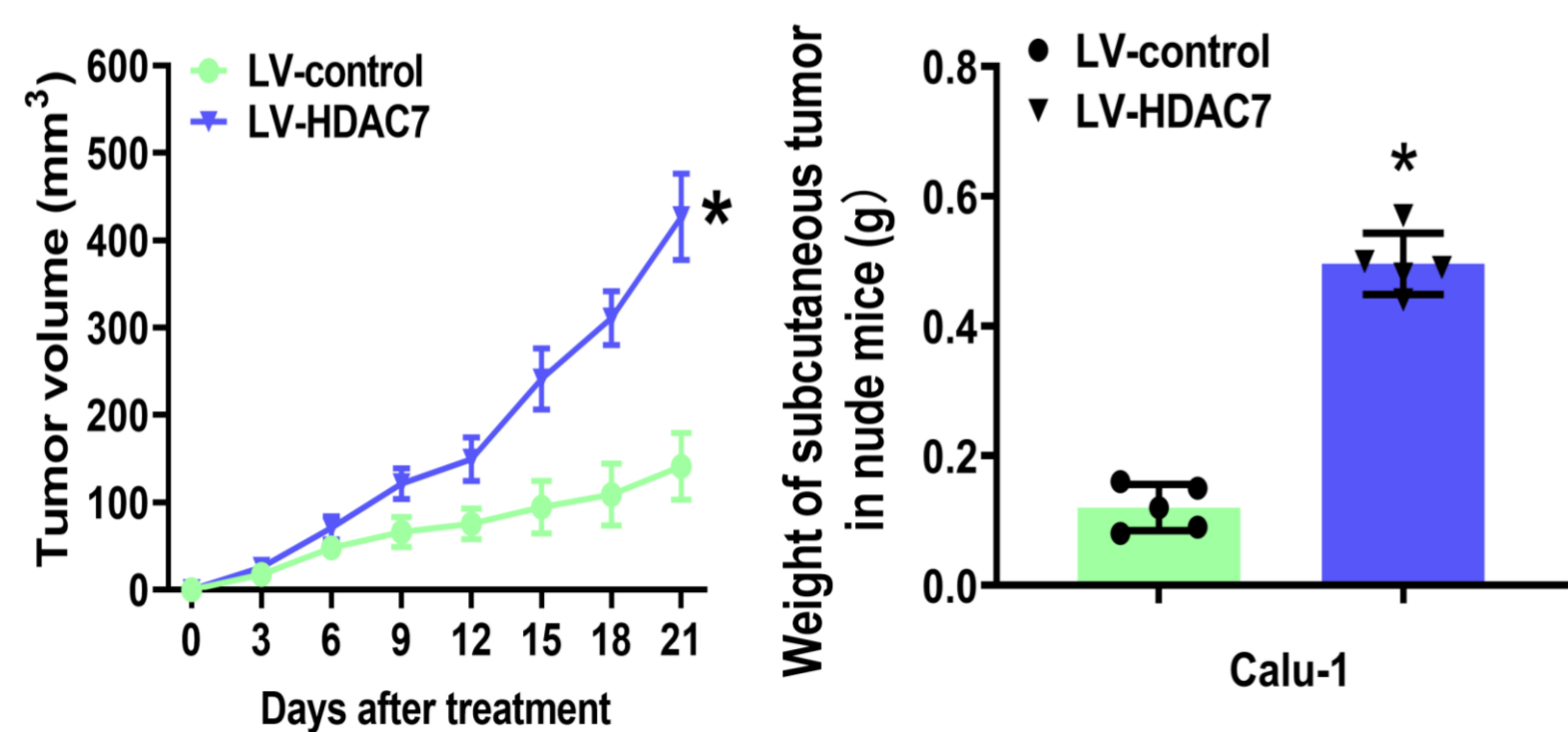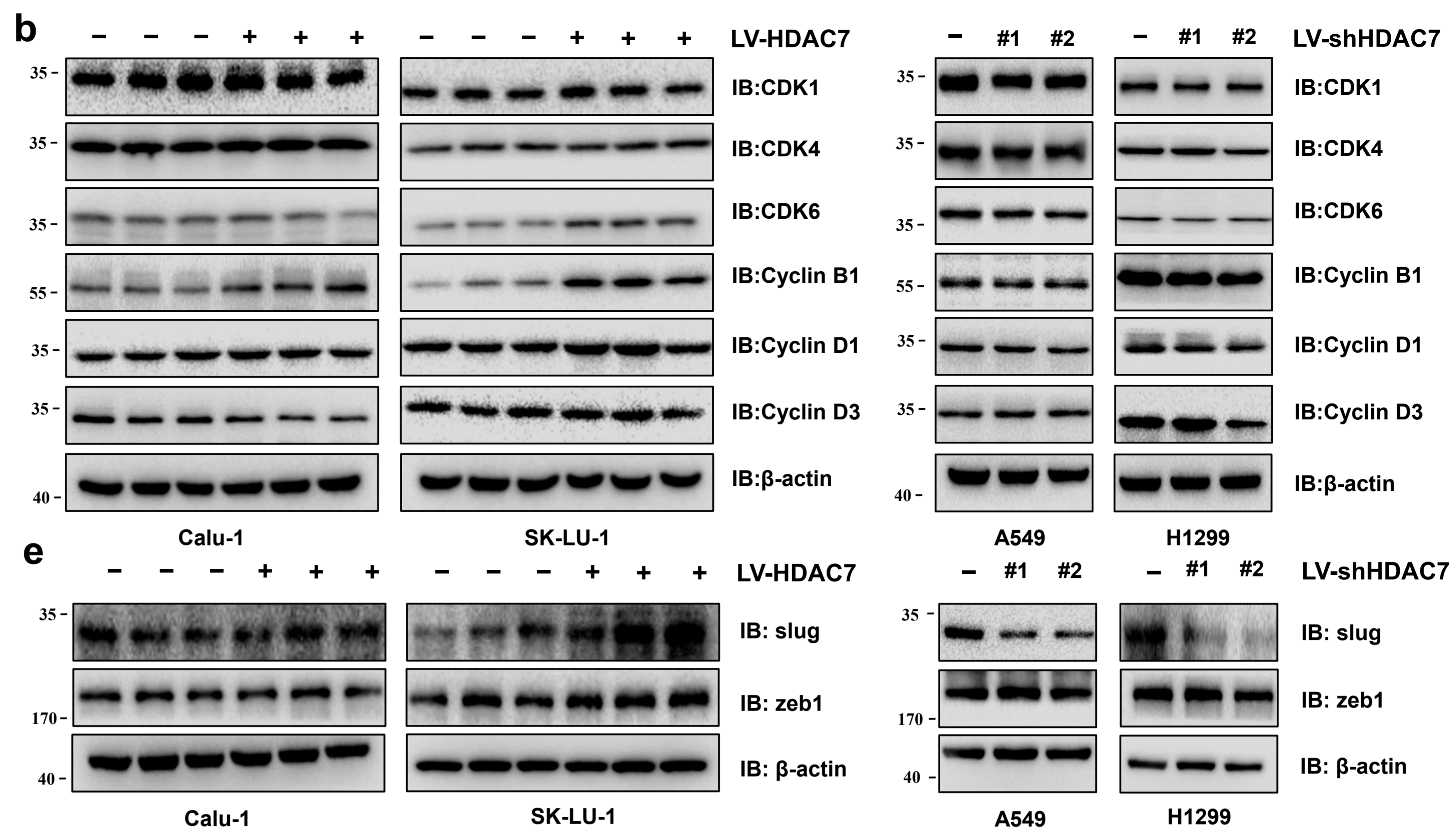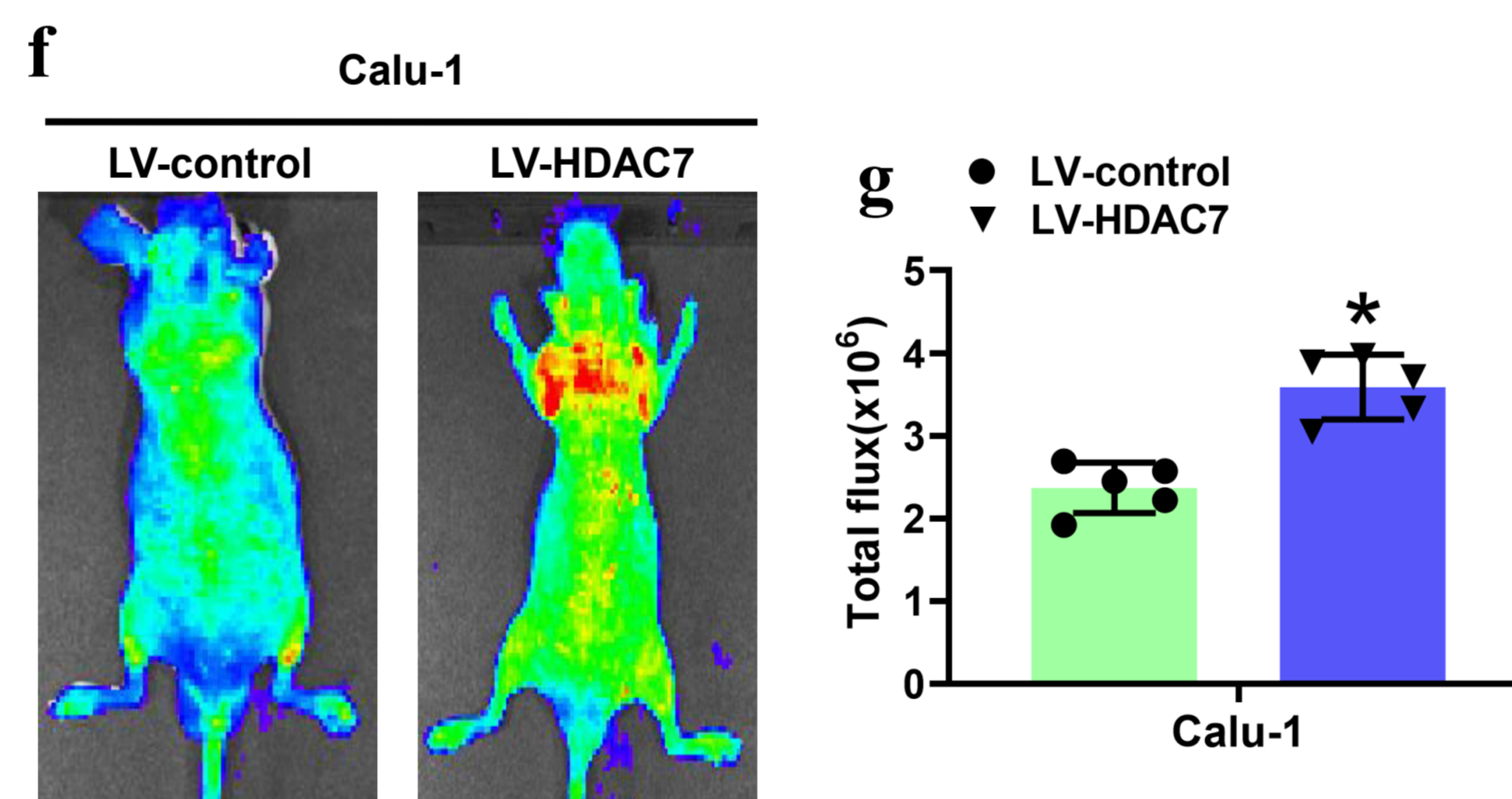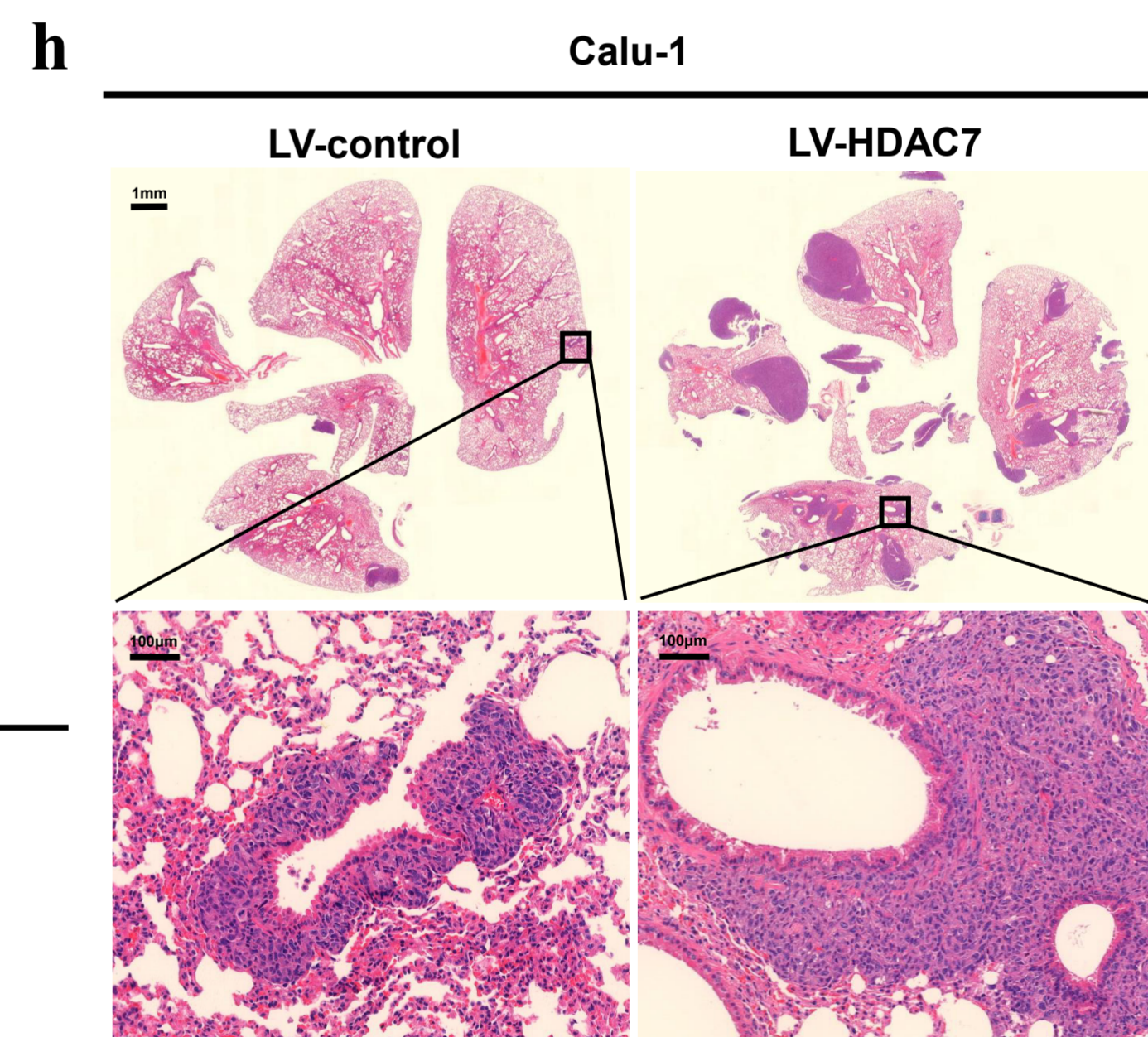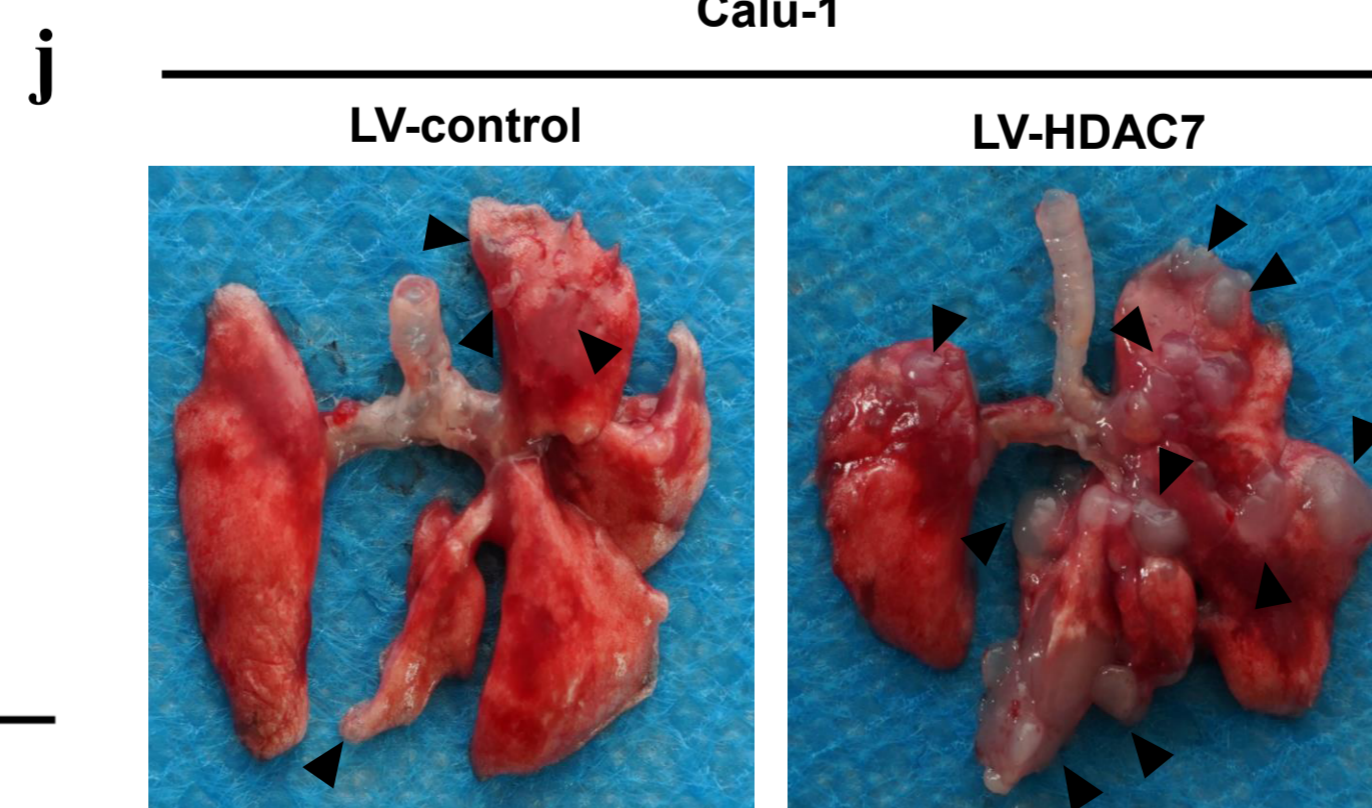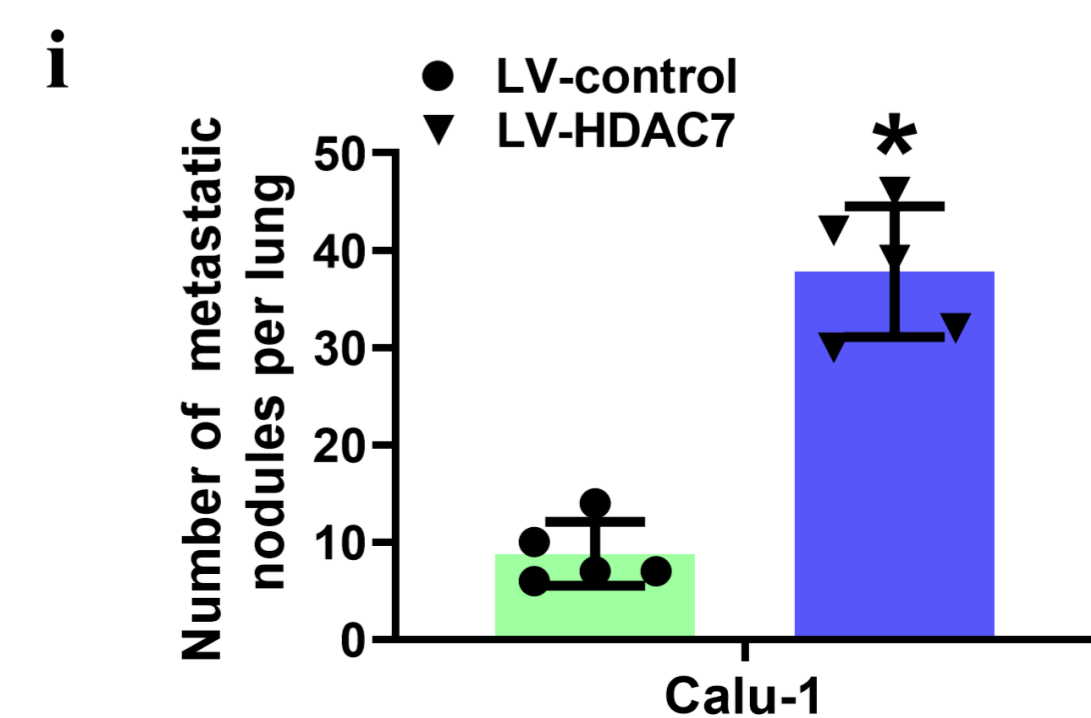

Supplement: Supplementary file 1 — Additional file 1: Figure 1. (a) The basal HDAC7 expressions of NSCLC cell lines. (b) Representative western blotting of cell cycle–related proteins in HDAC7 overexpression and knockdown cells. (c) Flow cytometry results of cell-cycle distribution at indicated time points. (d) Gross photograph of subcutaneous xenograft tumors, tumor growth curve and subcutaneous tumor weight in each group. (e) Representative western blot of EMT-associated proteins in HDAC7 overexpression and knockdown cells. Representative fluorescence images (f) and fluorescence signals analysis (g) of pulmonary metastases 4 weeks after tail vein injection. (h) Representative HE staining images of lung samples from indicated groups. Scale bar, 1 mm and 100 μm (inset), respectively. (i) The number of metastatic nodules per lung in HE staining images. (j) Representative general morphology of surface lung metastases. The black triangular arrows indicate the metastatic nodules. β-actin was used as internal control. All the data are expressed as mean ± SD. *P < 0.05. LV, lentivirus. [file 13046_2022_2266_MOESM1_ESM.pdf]

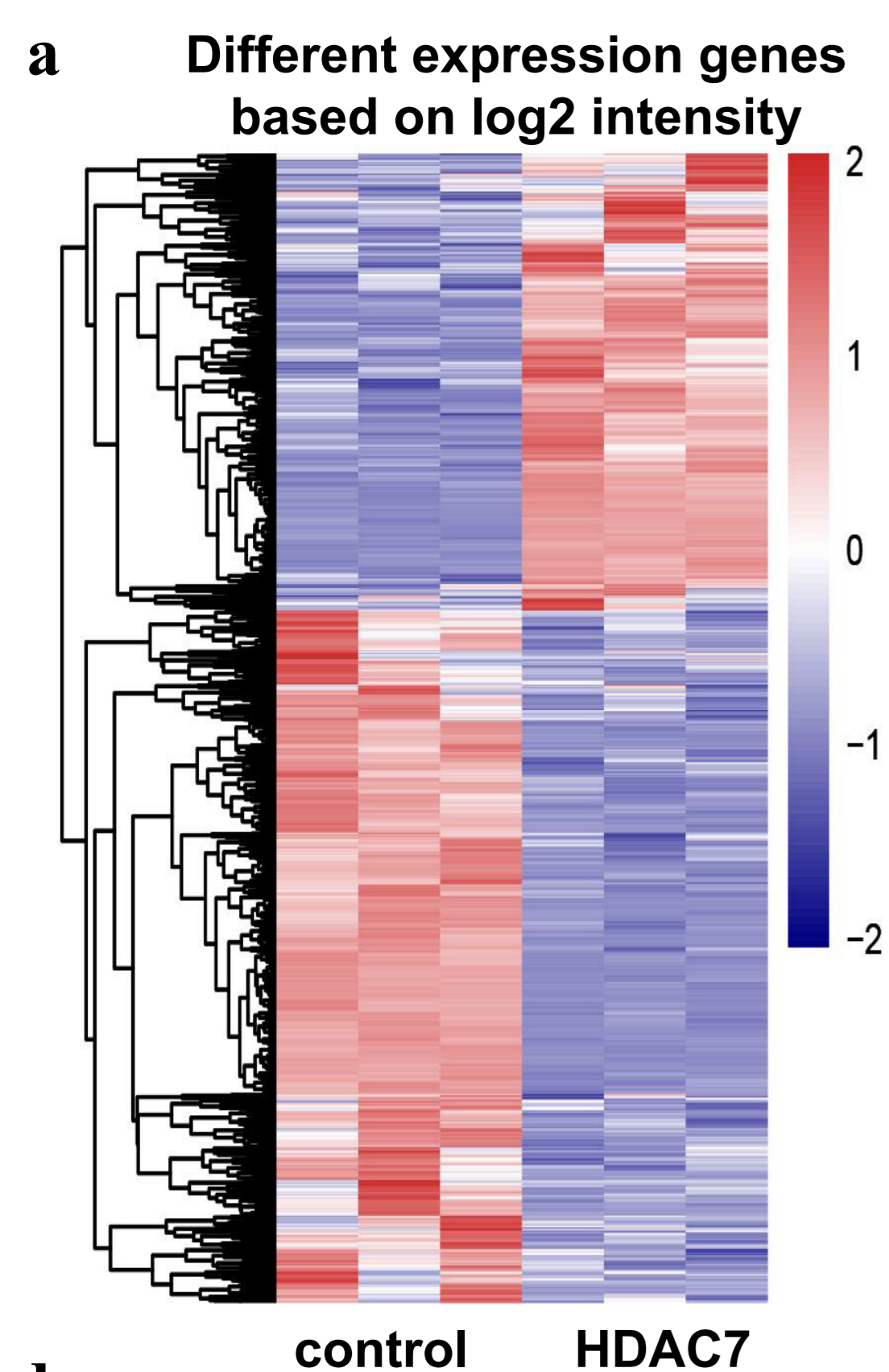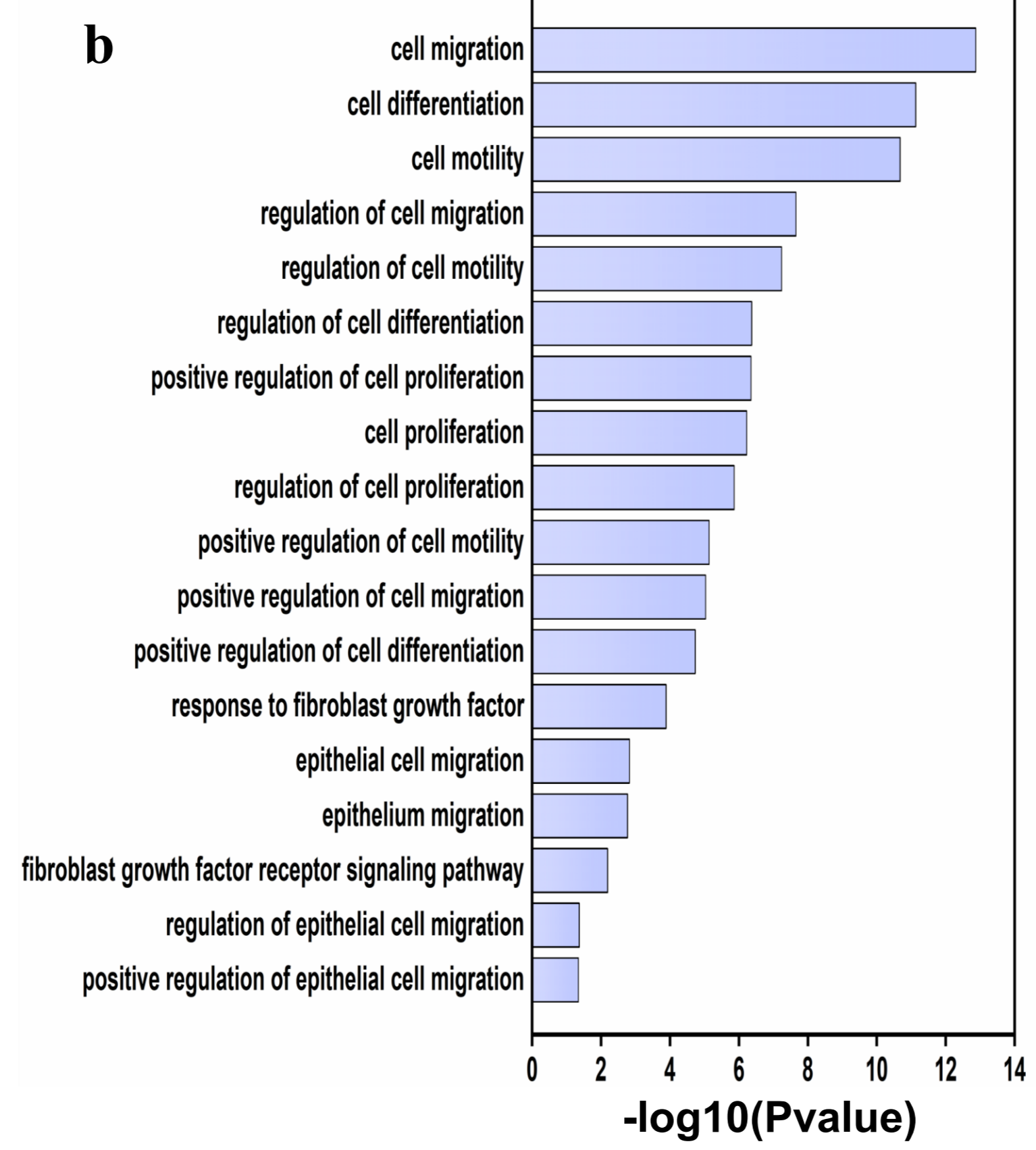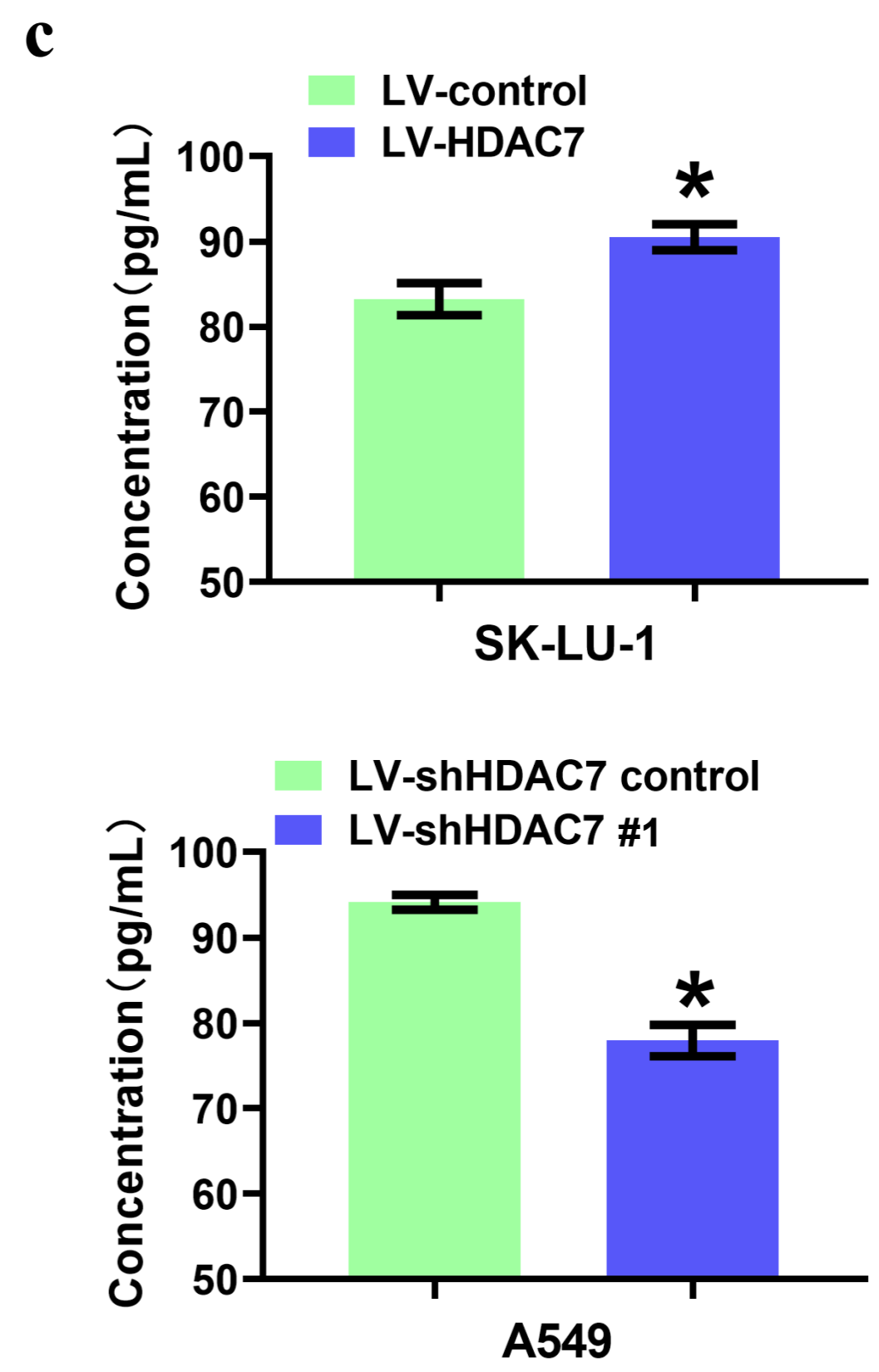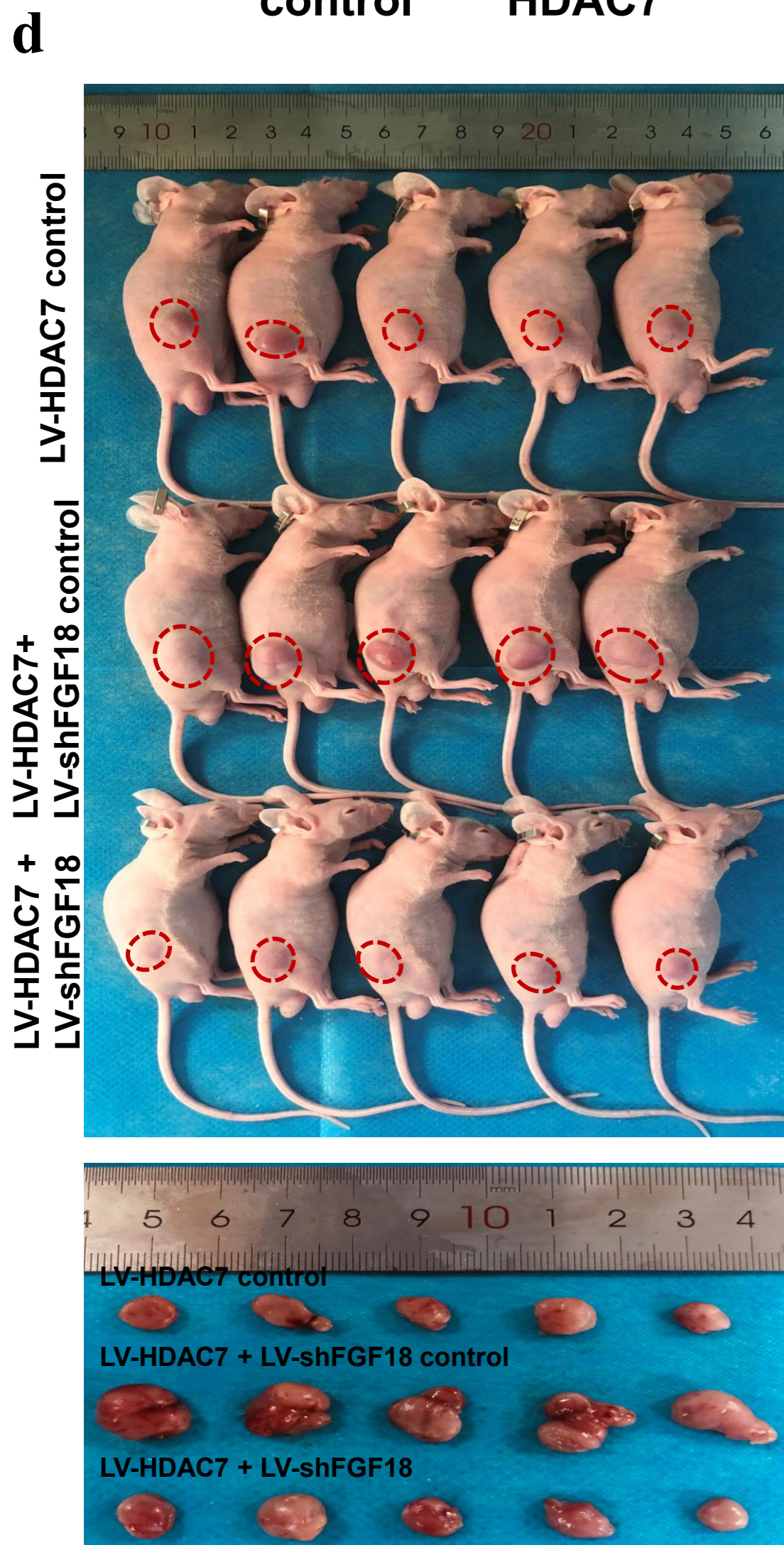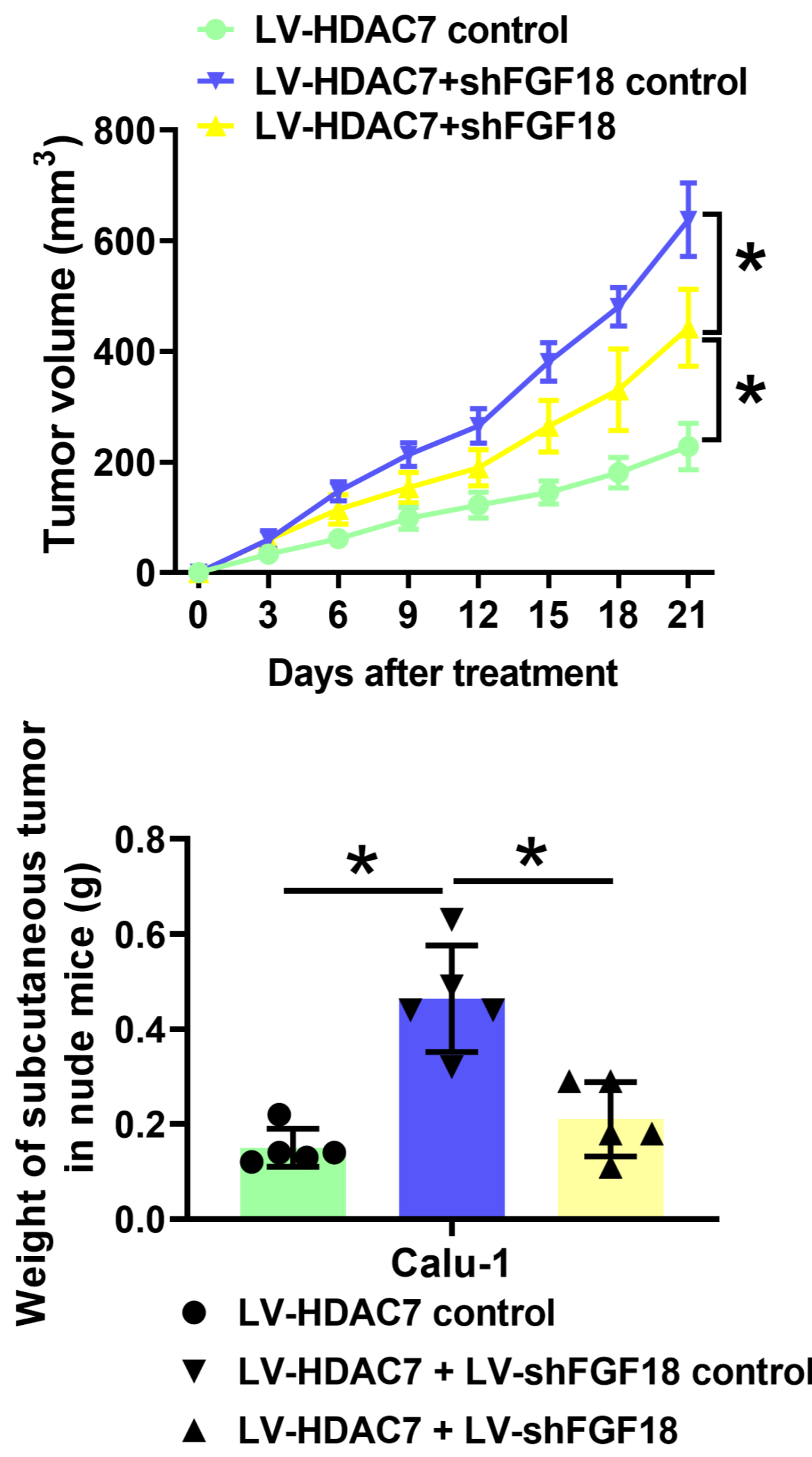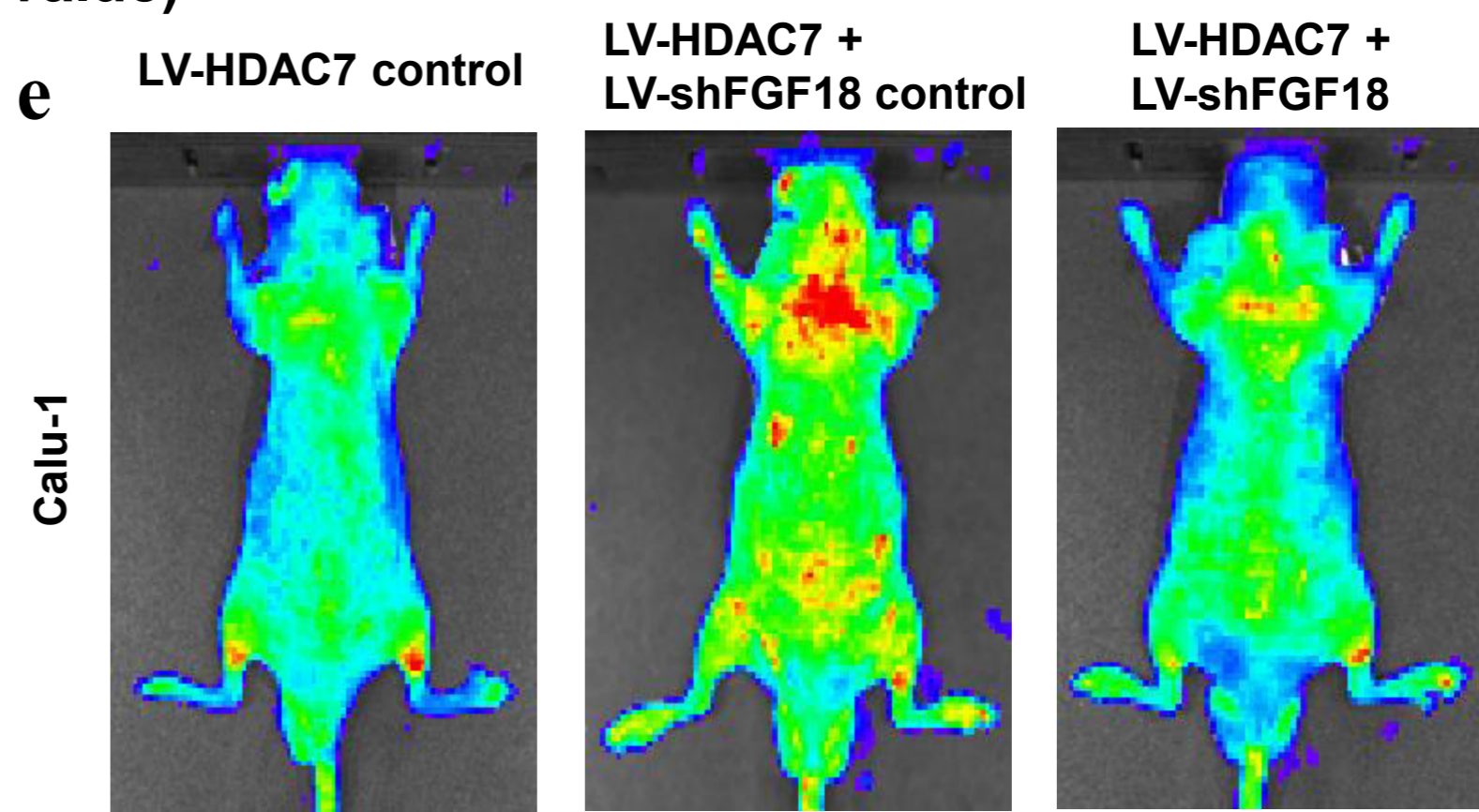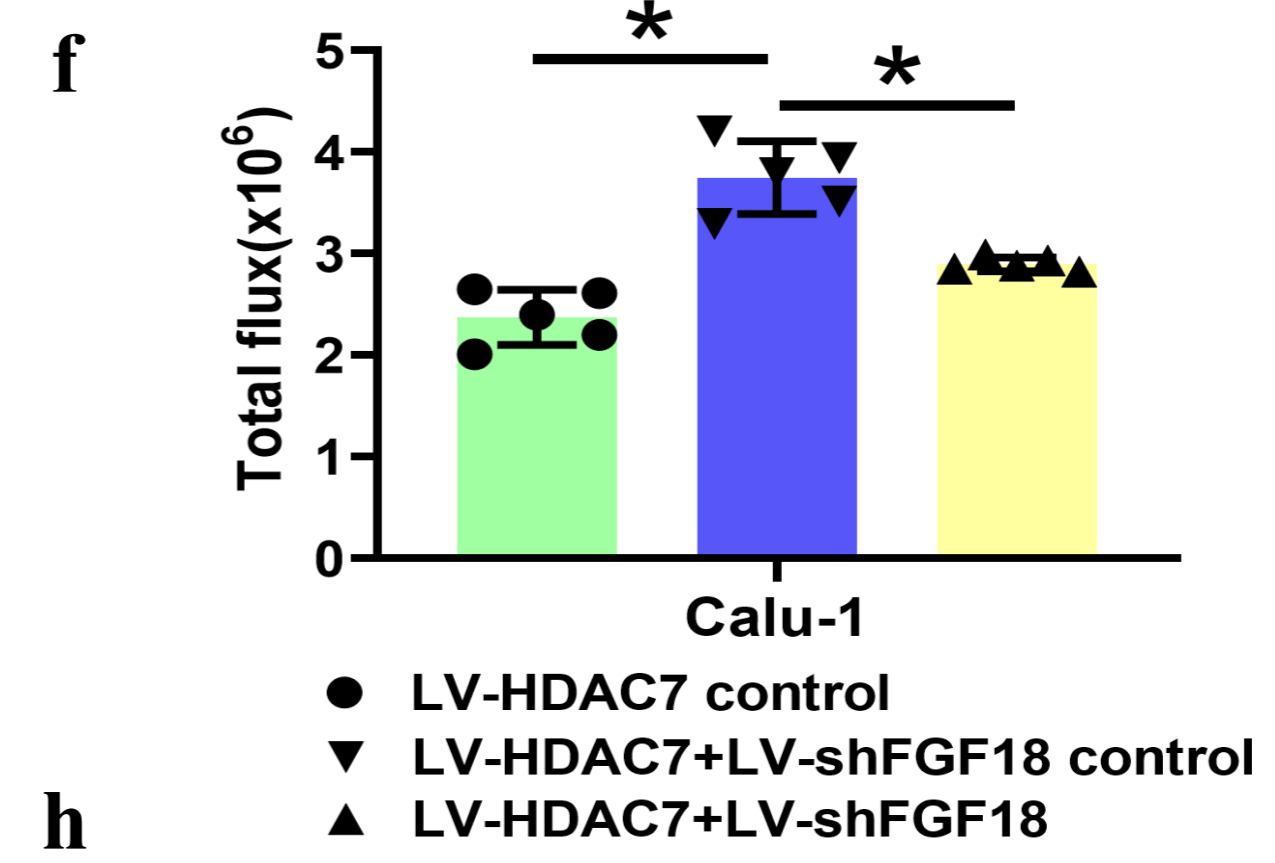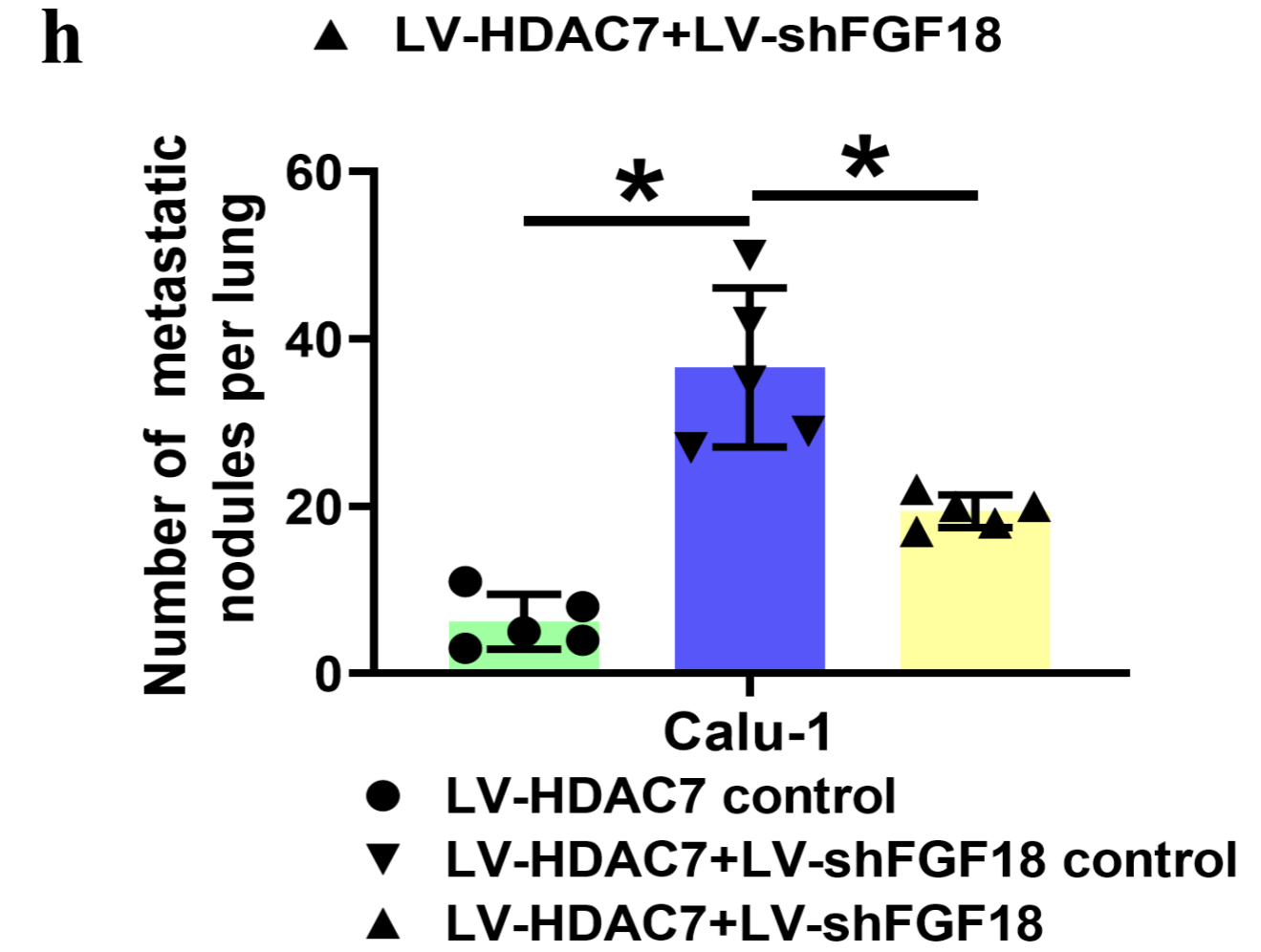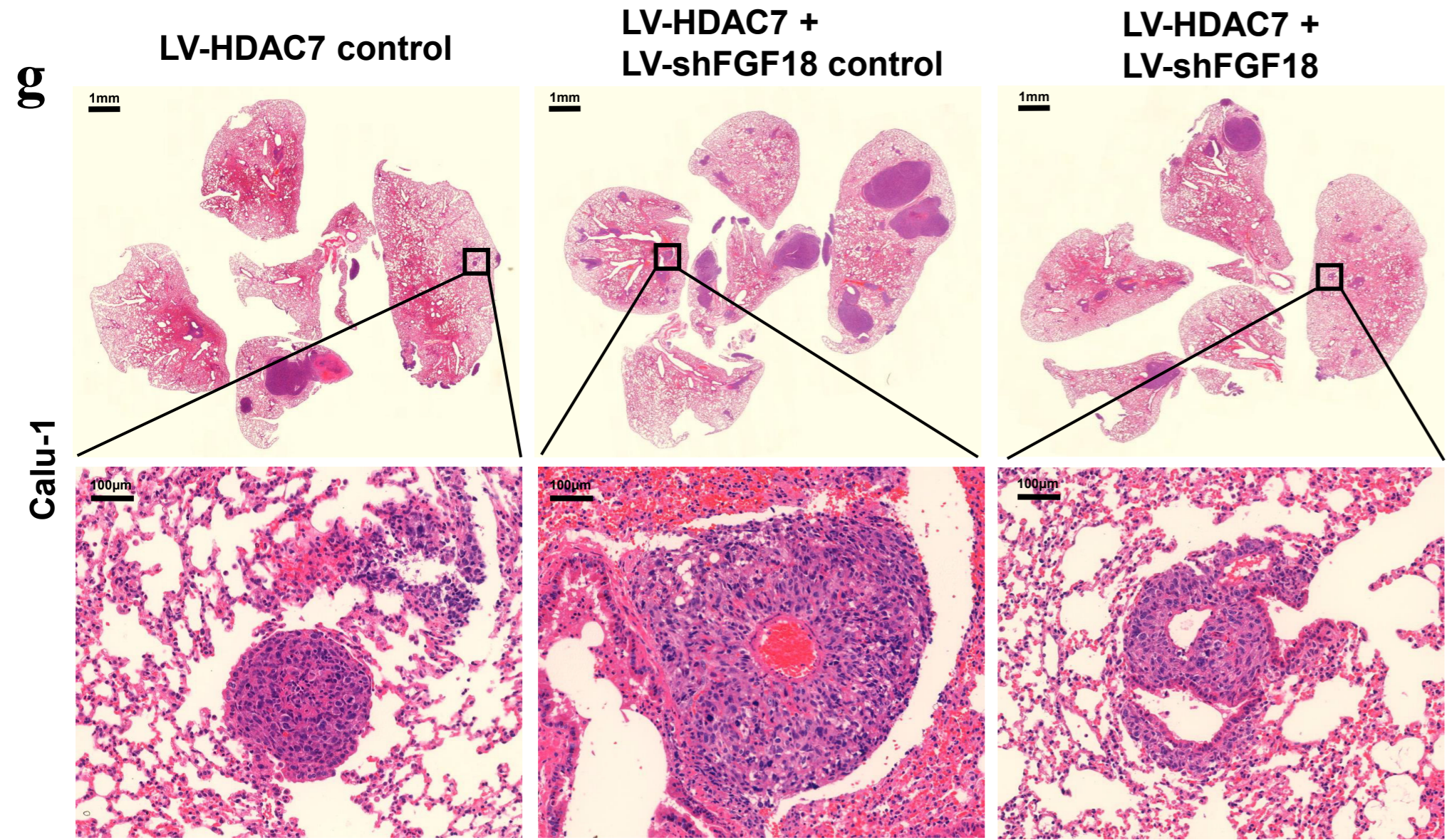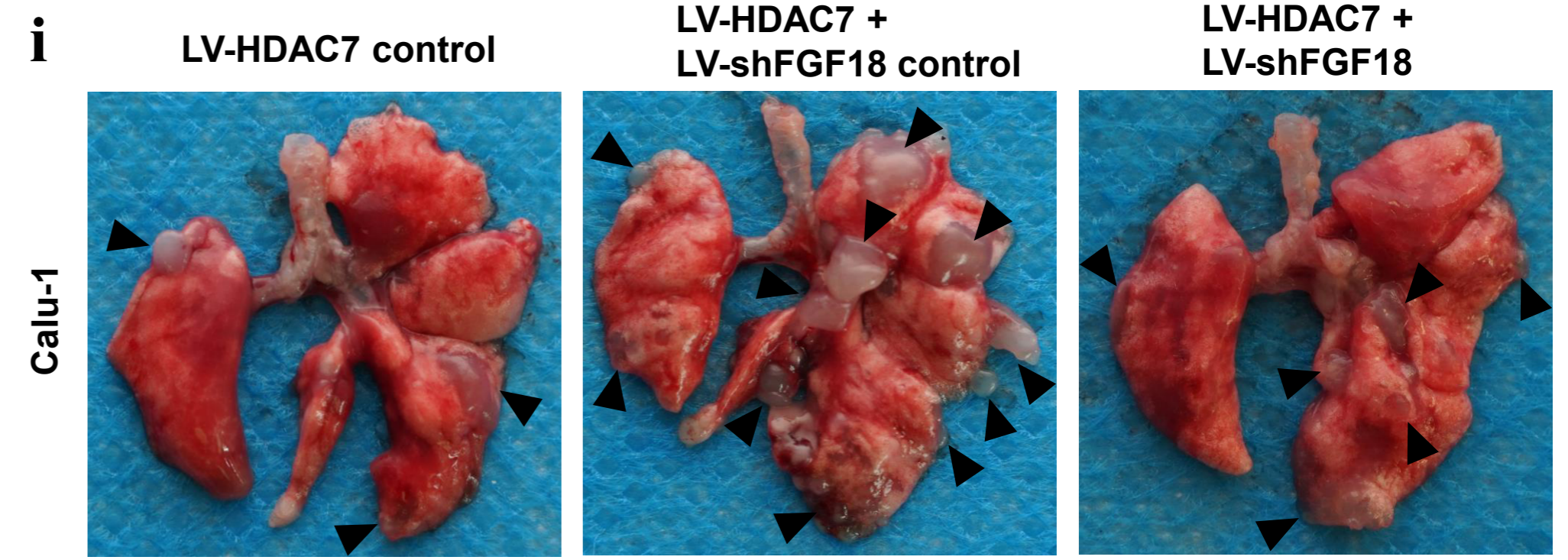

Supplement: Supplementary file 2 — Additional file 2: Figure 2. (a) Heatmap of RNA-seq data from SK-LU-1 cells overexpressing HDAC7 or the control based on the log2 intensity. (b) The GO function enrichment analysis of DEGs in SK-LU-1 cells overexpressing HDAC7 or the control, ranked by the P value. (c) The quantification changes of FGF18 in the culture medium after up- or downregulating HDAC7 in NSCLC cells. (d) Gross photograph of subcutaneous xenograft tumors, tumor growth curve and tumor weight in indicated groups. Representative fluorescence images (e) and fluorescence signals analysis (f) of pulmonary metastases 4 weeks after tail vein injection. (g) Representative HE staining images of lung samples from indicated groups. Scale bar, 1 mm and 100 μm (inset), respectively. (h) The number of metastatic nodules per lung in HE staining images. (i) Representative general morphology of surface lung metastases. The black triangular arrows indicate the metastatic nodules. All the data are expressed as mean ± SD. *P < 0.05. LV, lentivirus. [file 13046_2022_2266_MOESM2_ESM.pdf]
