# Supplementary material for: HDAC7 promotes NSCLC proliferation and metastasis via stabilization by deubiquitinase USP10 and activation of β-catenin-FGF18 pathway
Source: J Exp Clin Cancer Res. 2022 Mar 11;41:91. doi: 10.1186/s13046-022-02266-9 (PMC8915541; doi:10.1186/s13046-022-02266-9)
Supplement: Supplementary file 3 — Additional file 3: Figure 3. (a) The relative TOP/FOP ratio in HDAC7 overexpression or knockdown NSCLC cells. The mRNA (b) and protein levels (c) of β-catenin related genes in HDAC7 overexpression or knockdown NSCLC cells. (d) Representative western blotting of HDAC7-β-catenin-FGF18 pathway involved proteins in indicated NSCLC cells. (e) Mass spectrometry analysis results of USP10 peptide sequence (IAELLENVTLIHKPVSLQP). (f) Representative HDAC7 and USP10 IHC images of NSCLC and adjacent non-tumor tissues. Scale bar, 200 μm and 50 μm (inset), respectively. Correlation analysis of HDAC7 and USP10 expression in NSCLC tissues (g) and in adjacent non-tumor tissues (h). β-actin was used as internal control. All the data are expressed as mean ± SD. *P < 0.05. LV, lentivirus. [file 13046_2022_2266_MOESM3_ESM.pdf]

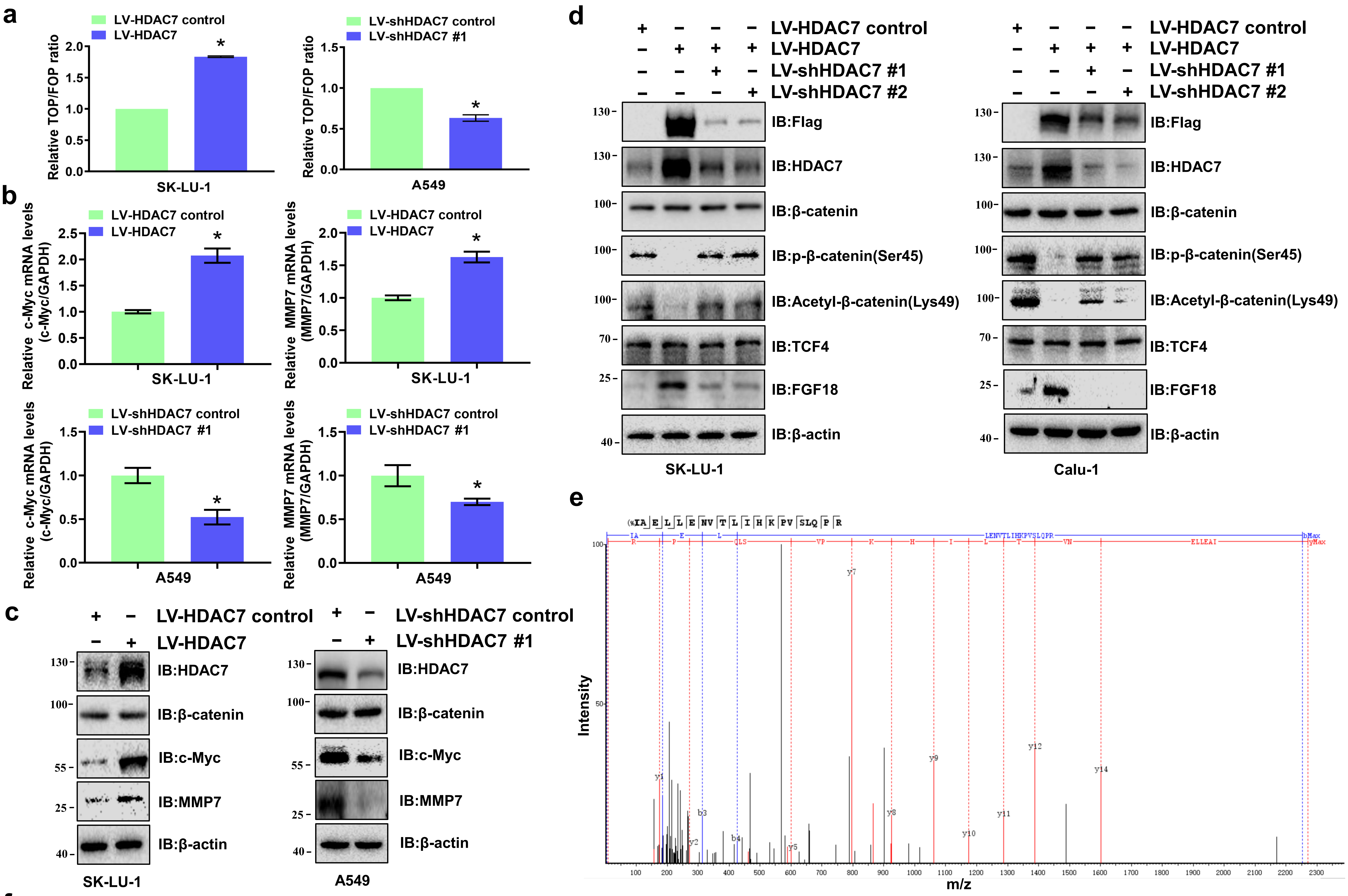

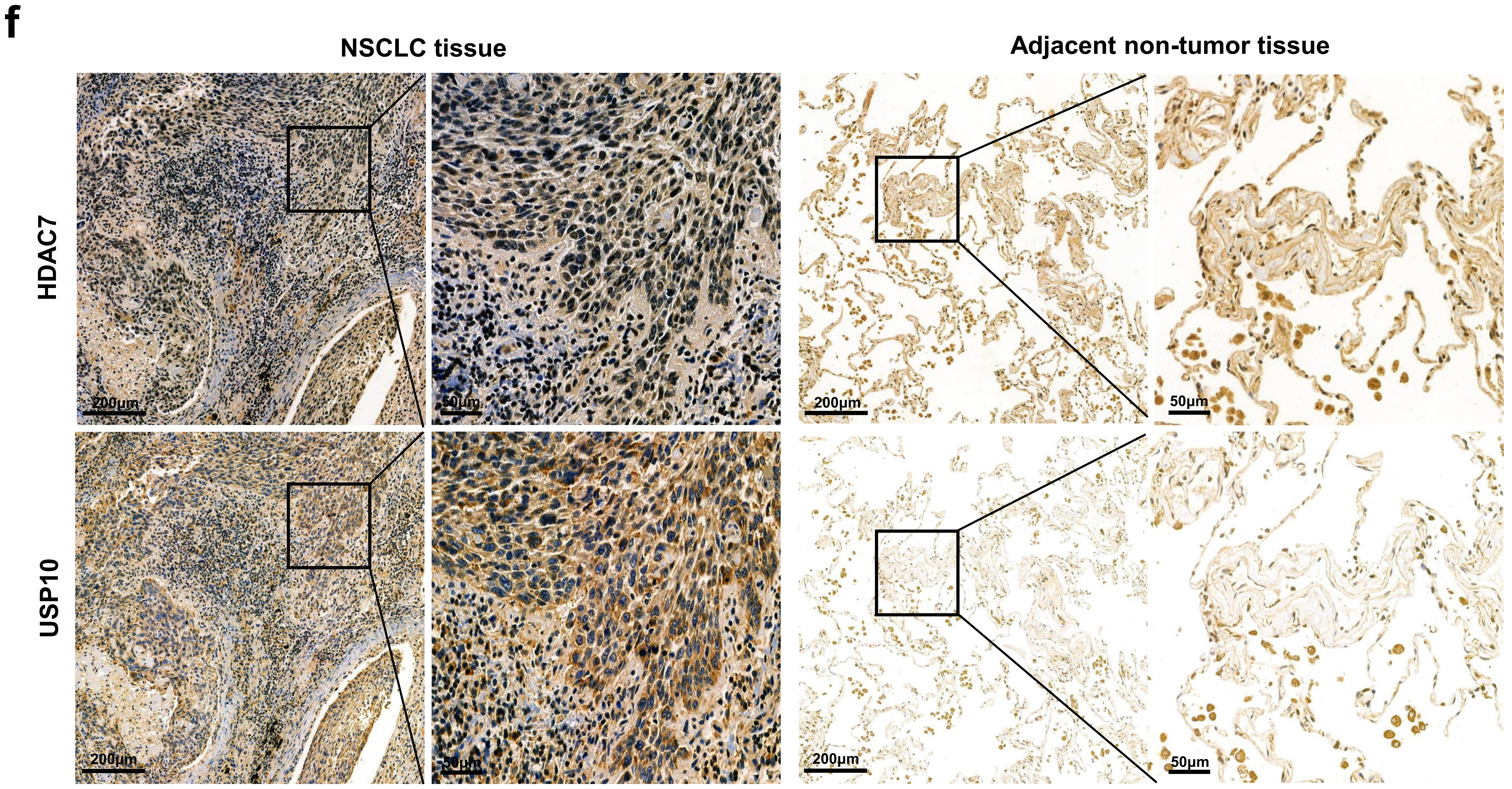

g

Correlation analysis of HDAC7 and USP10 Expression in NSCLC tissues (N=71)

		USP10 High (N=35)	USP10 Low (N=36)	Correlation coefficient	p value
HDAC7	High (N=26)	21	5	0.479	< 0.001
	Low (N=45)	14	31		

h

Correlation analysis of HDAC7 and USP10 Expression in adjacent non-tumor tissues (N=71)

		USP10 High (N=30)	USP10 Low (N=41)	Correlation coefficient	p value
HDAC7	High (N=14)	4	10	-0.137	0.254
	Low (N=57)	26	31		
